# PRIME 2.0: Proposed Requirements for Cardiovascular Imaging-Related Multimodal-AI Evaluation: An Updated Checklist

**DOI:** 10.1016/j.jcmg.2025.08.004

**Published:** 2025-08-27

**Authors:** Nobuyuki Kagiyama, Márton Tokodi, Quincy A. Hathaway, Rima Arnaout, Rhodri Davies, Damini Dey, Nicolas Duchateau, Alan G. Fraser, Shinichi Goto, Ankush D. Jamthikar, Carolyn S.P. Lam, Evangelos K. Oikonomou, David Ouyang, Ambarish Pandey, Timothy J. Poterucha, Zahra Raisi-Estabragh, Jordan B. Strom, Qiang Zhang, Naveena Yanamala, Partho P. Sengupta

**Affiliations:** a Department of Cardiovascular Biology and Medicine, Juntendo University Graduate School of Medicine, Tokyo, Japan; b AI Incubation Farm, Juntendo University Faculty of Medicine, Tokyo, Japan; c Heart and Vascular Center, Semmelweis University, Budapest, Hungary; d Department of Experimental Cardiology and Surgical Techniques, Semmelweis University, Budapest, Hungary; e Department of Radiology, University of Pennsylvania, Philadelphia, Pennsylvania, USA; f Department of Medicine, Radiology, and Pediatrics, University of California, San Francisco, California, USA; g Bakar Institute, University of California, San Francisco, California, USA; h UCSF-UC Berkeley Joint Program in Computational Precision Health, San Francisco, California, USA; i Institute of Cardiovascular Science, University College London, London, United Kingdom; j Cedars-Sinai Medical Center, Biomedical Sciences, Los Angeles, California, USA; k Universite Claude Bernard Lyon 1, INSA-Lyon, CREATIS lab, CNRS UMR5220, Inserm U1294, Villeurbanne, France; l Institut Universitaire de France (IUF), Paris, France; m School of Medicine, Cardiff University, Cardiff, United Kingdom; n Division of Cardiovascular Medicine, Brigham and Women’s Hospital, Harvard Medical School, Boston, Massachusetts, USA; o Department of Medicine, Tokai University School of Medicine, Japan; p Division of Cardiovascular Diseases and Hypertension, Department of Medicine, Rutgers Robert Wood Johnson Medical School, New Brunswick, New Jersey, USA; q National Heart Centre Singapore and Duke-National University of Singapore, Singapore; r Section of Cardiovascular Medicine, Department of Internal Medicine, Yale School of Medicine, New Haven, Connecticut, USA; s Department of Cardiology, Smidt Heart Institute, Cedars-Sinai Medical Center, Los Angeles, California, USA; t Division of Research, Kaiser Permanente Northern California, Pleasanton, California, USA; u Division of Cardiology, Department of Internal Medicine, University of Texas Southwestern Medical Center, Dallas, Texas, USA; v Mayo Clinic, Division of Echocardiography and Cardiac Imaging, Department of Cardiology, Rochester, Minnesota, USA; w Division of Cardiology, Department of Medicine, Columbia University, New York, New York, USA; x William Harvey Research Institute, NIHR Barts Biomedical Research Centre, Queen Mary University of London, Charterhouse Square, London, United Kingdom; y Richard A. and Susan F. Smith Center for Outcomes Research in Cardiology, Boston, Massachusetts, USA; z Division of Cardiovascular Medicine, Beth Israel Deaconess Medical Center, Boston, Massachusetts, USA; aa Harvard Medical School, Boston, Massachusetts, USA; bb Division of Cardiovascular Medicine, Radcliffe Department of Medicine, University of Oxford, Oxford, United Kingdom; cc Big Data Institute, Li Ka Shing Centre for Health Information and Discovery, University of Oxford, Oxford, United Kingdom.

**Keywords:** artificial intelligence, cardiovascular imaging, clinical validation, deep learning, large language models, model development, multimodal generative artificial intelligence, PRIME 2.0 checklist, transparency and reproducibility

## Abstract

The PRIME (Proposed Requirements for Cardiovascular Imaging–Related Machine Learning Evaluation) 2.0 checklist is an updated, domain-specific framework designed to standardize the development, evaluation, and reporting of artificial intelligence (AI) applications in cardiovascular imaging. This update specifically responds to rapid advances from traditional machine learning to deep learning, large language models, and multimodal generative AI. The updated checklist was developed through a modified Delphi process by an international panel of clinical and technical experts. In contrast to general AI reporting guidelines, it delivers detailed, practical recommendations on all critical aspects of AI research and builds upon the original 7-domain framework by incorporating cardiovascular imaging–specific complexities such as cardiac motion, imaging artifacts, and interobserver variability. By promoting transparency and rigor, PRIME 2.0 can serve as a vital resource for researchers, clinicians, peer reviewers, and journal editors working at the forefront of AI in cardiovascular imaging.

## CENTRAL ILLUSTRATION

The PRIME 2.0 Framework and Checklist for Developing and Reporting AI Studies in Cardiovascular Imaging

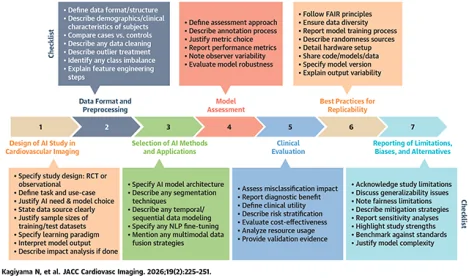

The PRIME (Proposed Requirements for Cardiovascular Imaging-Related Multimodal-AI Evaluation) 2.0 framework, which guides researchers through 7 key domains in the lifecycle of artificial intelligence (AI) studies in cardiovascular imaging: study design, data preprocessing, AI method selection, model assessment, clinical evaluation, replicability practices, and reporting of limitations. Each domain includes a detailed checklist of best practices to promote transparency, reproducibility, and clinical relevance in AI research. FAIR = findable, accessible, interoperable, reusable; NLP = natural language processing; RCT = randomized controlled trial.

## RATIONALE FOR PRIME 2.0: RESPONDING TO A RAPIDLY EVOLVING ARTIFICIAL INTELLIGENCE LANDSCAPE

In 2020, the PRIME (Proposed Requirements for Cardiovascular Imaging-Related Machine Learning Evaluation) checklist was published in *JACC: Cardiovascular Imaging*.^1^ The checklist provided a critical framework for standardizing the quality and reporting of research within the cardiovascular imaging community when machine learning (ML) was performed mostly using tabular data. However, the landscape of artificial intelligence (AI) in medicine has transformed at a dramatic pace. Since 2020, publications featuring “deep learning” and “cardiovascular imaging” have grown exponentially.^2^ The research frontier has rapidly progressed from using tabular data to analyzing raw pixel data with deep learning (DL)^3^ and using generative models for synthetic data creation.^4^ It is now advancing toward large language models (LLMs) and multimodal AI, which can integrate imaging data with clinical notes, electronic health records (EHRs), and genomic data.^5^ Recognizing that this technological evolution demands updated standards, *JACC: Cardiovascular Imaging* invited a new expert panel to develop PRIME 2.0.

## COMPARISON WITH OTHER GUIDELINES AND THE UNIQUE NEEDS OF AI IN CARDIOVASCULAR IMAGING

A landscape of reporting guidelines, summarized in Supplemental Table 1, has been established to improve the transparency of AI research, including the well-known recommendations such as TRIPOD (Transparent Reporting of a Multivariable Prediction Model for Individual Prognosis or Diagnosis) + AI.^6^ PRIME 2.0 is not intended to replace these but to serve as a complementary, domain-specific standard. AI research in cardiovascular imaging faces unique challenges that are not fully addressed by the general guidelines. These include the dynamic motion of the heart, high anatomical variability, significant interobserver variability in “ground-truth” annotations, and modality-specific artifacts and noise. Table 1 summarizes these key issues in cardiovascular imaging AI research that this guideline aims to help researchers address in a systematic manner.

## METHODOLOGY FOR THE UPDATE PROCESS

*JACC: Cardiovascular Imaging* invited an international, multidisciplinary expert panel comprising clinicians, computer scientists, and data scientists with expertise in both cardiovascular imaging and AI. The panel used a modified Delphi method,^7^ engaging in iterative rounds of reviewing evidence, drafting a checklist, and providing anonymous feedback. This consensus-driven approach was used to refine each recommendation and ensure that the final checklist reflects the state-of-the-art and practical needs of the research community. All invited experts made substantive contributions to both the review of published reports and manuscript development. Dr Sengupta coordinated the process, organizing 3 working groups led by Drs Kagiyama, Tokodi, and Hathaway, each combining AI engineers/data scientists with physician-scientists. After initial planning meetings, members were assigned specific topics with defined word limits, checklist elements, and table content. They independently conducted searches of published reports and drafted sections, which were compiled into an initial draft. Dr Sengupta and the group leaders then led multiple collaborative editing sessions to refine the text and checklists, followed by circulation to all authors for review. Blinded survey and voting were used to reach the final consensus, ensuring that all authors met established authorship criteria for the main manuscript.

## OVERVIEW OF THE PRIME 2.0 FRAMEWORK AND CHECKLIST STRUCTURE

The PRIME 2.0 framework is structured to guide researchers through the entire lifecycle of an AI study. It expands upon the 7 core domains established in the original version (Central Illustration), with substantially revised content to provide granular guidance on contemporary AI methodologies and the unique challenges of cardiovascular imaging. This effort culminates in a comprehensive checklist (Table 2). The checklist follows a tiered structure, with essential items marked by a green checkbox and optional items by a grey checkbox. Furthermore, 2 independent checklist templates have been provided for researchers (Supplemental Essential Checklist Template, Supplemental Extended Checklist Template). To promote transparency and scientific rigor, we encourage authors to complete and submit these essential items when reporting on AI in cardiovascular imaging. The remaining, nonhighlighted items are also desirable, and we strongly encourage their inclusion in manuscripts whenever feasible. However, we acknowledge that the nonhighlighted items are resource intensive and may not always be pursued, particularly in smaller clinical or academic settings.

## TARGET AUDIENCE AND SCOPE OF THE DOCUMENT

The intended audience for PRIME 2.0 can be divided into 3 groups. It is designed primarily for investigators and data scientists conducting research at the intersection of AI and cardiovascular imaging. Secondly, it can serve as a resource for clinicians, trainees, and other readers who must critically appraise the findings of these complex studies. Finally, it provides a structured framework for peer reviewers and journal editors to assess the rigor, validity, and transparency of submitted manuscripts. To enhance readability for a clinical audience while preserving the necessary technical depth, the document presents general principles in the main text. Specifically, during the manuscript development process, authors have contributed detailed technical content for later sections “Selection of AI Methods and Applications,” “Model Assessment,” and “Clinical Evaluation.” To maintain clarity and narrative flow for a clinical audience, this content was relocated to dedicated panels. References for the technical content are provided in the Supplemental References. Recommendations related to regulatory approval, health technology assessment, and post-market surveillance and clinical follow-up will be addressed in later publication.

## DESIGNING AN AI STUDY IN CARDIOVASCULAR IMAGING

### APPROPRIATENESS OF APPLYING AI.

The availability of AI has supported many advances in data-driven health care over the past 2 decades. At the same time, there remain challenges that are difficult for even the most advanced AI techniques to solve. An important first task is determining whether input data are available, suitable, and of sufficient scale. However, the input data may be uninformative^8^ or confounded,^9^ making it a challenging substrate to train efficient AI models. Similarly, it is well known that the scale of training data greatly impacts model performance.^10^ An implicit corollary would then mean that a model trained, eg, on historical echocardiogram images (of which we have many years of stored data to work with) would outperform a model trained on point-of-care ultrasound data (as images acquired during such studies are frequently not saved and limited to more recent years). If applicable, researchers should also justify why they expect complex AI models to substantially outperform simpler ML or conventional statistical approaches, especially when using tabular data as input.

### STUDY OBJECTIVES, INPUT DATA TYPE, AND PREDICTION TARGET.

Studies featuring AI methods should clearly define the objectives, planned deployment context, input data, and desired output. These elements establish the scope of the problem and the data sources that inform the model design principles. The study’s objective is framed by the desired clinical task and deployment context. These elements account for the clinical problem that will be addressed and the intended user and clinical workflows in which an AI-based solution is anticipated to be deployed. The model development strategy, which could be either the development of a new model (ie, de novo model development), an adaptation or fine-tuning of an existing model, or the external validation of a previously published model, should be specified. It should also be clearly stated whether the investigators aim to develop a foundation model, which is a model trained on large, diverse data sets with the goal of being adaptable to a wide range of downstream tasks.

The input modality refers to the type of data used to train the model. For image-based models, key details such as dimensionality, imaging modality, data source, and acquisition parameters should be clearly described. The supervision type defines the learning paradigm: supervised learning (with labeled outputs), unsupervised learning (pattern discovery without labels), or hybrid approaches that combine elements of both. The model output type describes the nature of the predictions, such as binary or multiclass classification, continuous values, or clustering. Supplemental Table 2 presents these elements in the context of 3 published studies.

The transparent description of the data used in AI systems is essential for reproducibility, bias detection and mitigation, gaining user trust, and satisfying ethical and regulatory requirements. A detailed description can also address difficulties in standardizing imaging protocols across centers. The type of input data should be described (eg, cardiac images), structured data (eg, vital signs, laboratory results, and medication lists), unstructured data (eg, clinical notes, clinic letters, and radiology reports), and continuous signal data (eg, electrocardiogram [ECG] or data from wearables). If images are used, the dimensionality (2-dimensional, 3-dimensional, ± time), modality (x-ray, computed tomography, magnetic resonance imaging [MRI], ultrasound, or nuclear imaging), and data source (such as, scanner type, make, model, and software version) should be rigorously reported. The nature of the model’s prediction (eg, probability, classification, segmentation, generative image), what it represents (eg, the probability of a disease), and—if applicable—an estimate of uncertainty should all be documented.

### DESIGN OF THE AI STUDY.

AI study designs may be categorized as: 1) retrospective analysis of previously collected data;^11^ 2) prospective data collection enabling the documentation of all suspected confounders;^12,13,14^ 3) a combination of retrospective analysis and prospective data collection; 4) post hoc analysis of a randomized controlled trial (RCT) or a registry;^15^ or 5) an evaluation within a prospective RCT^16^ (Table 3). In addition to traditional RCTs, new trial designs such as pragmatic RCTs^17^ and hybrid effectiveness-implementation trials^18^ are increasingly used to evaluate AI applications in real-world settings. Pragmatic RCTs assess whether AI works in routine practice rather than under ideal conditions. Designs such as cluster RCTs (randomizing facilities)^19^ and stepped-wedge cluster RCTs (gradually introducing AI to all sites)^20^ may help reduce contamination. Randomized crossover trials are also efficient,^21^ but researchers should be aware that clinician learning may lead to lasting carry-over effects.

Hybrid trials provide a more integrated approach by simultaneously evaluating clinical effectiveness and implementation strategies.^22^ These studies may primarily aim to determine the effectiveness of a clinical intervention (type 1), the utility of an implementation intervention (type 3), or equally address both aims (type 2). For example, type 2 trials may assess the diagnostic benefit of AI while comparing different training or workflow strategies to promote adoption.

The origin of the data should be specified as single-center or multicenter, clusters-based, and nation-wide or international. For clinical impact, the effect on the following criteria should be evaluated and reported, as applicable^23^: more precise patient selection; faster image acquisition; automated, faster, and more accurate post-processing and quantitative analysis; faster, more accurate diagnosis, prognosis, and intervention; improved clinical outcomes; and improved cost-effectiveness.

For study design, it is crucial to incorporate study endpoints and metrics to validate the standalone performance of the device as well as its performance within the intended clinical workflow while minimizing bias at all stages for improved generalizability.^23^

## DATA FORMAT AND PREPROCESSING

### DATA FORMAT.

Critical technical parameters must be reported for reproducibility, consistent with American Heart Association recommendations for detailed descriptions of data handling, including preprocessing, feature extraction, and transformations.^24,25^ For cardiovascular imaging studies, acquisition protocols such as ECG gating, breath-hold techniques, contrast timing, and standardized views (short-axis, 4-chamber, and so on) must be specified, along with imaging modality specifications (manufacturer, model, and acquisition software version; sequence parameters for MRI; tube current and slice thickness for computed tomography), image resolution, and file formats.^24,26^ Ensuring imaging data are well-characterized, representative, and reusable is essential for robust AI model development.^26^

We advocate using the term “reference standard” rather than “ground truth” to describe the benchmark against which model performance is measured, acknowledging inherent uncertainty in human annotations. Researchers should rigorously document the source of labels (expert clinicians, imaging reports, clinical outcomes), the number of annotators, and their qualifications, following recommendations for annotation using well-defined rules.^26^ Inter-rater reliability measures and consensus mechanisms for resolving disagreements should also be reported, when possible, as documentation of inter-rater variability is critical for assessing annotation quality.^24,26^ Researchers should also include the version of the annotation software, training materials provided to annotators, and quality control measures to ensure consistent data labeling across the data set, supporting the preparation of high-quality medical imaging data for DL applications.^25^

### CLINICAL CHARACTERISTICS OF THE STUDY COHORT.

Outlining the demographic and clinical characteristics of individuals in the study cohort is critical to ensuring the appropriate inclusion of under-represented patient groups and evaluating generalizability. Baseline characteristics of the study cohort should be reported in a table, presenting demographics, including age distribution, sex, race, and ethnicity, when feasible. Additional characteristics to be presented, when available, include anthropometrics (eg, body mass index), cardiovascular risk factors and other comorbidities, prior and current treatments (eg, medications, interventions), key vital parameters (eg, blood pressure, heart rate), and relevant imaging parameters (eg, left ventricular ejection fraction, ventricular volumes). When available, measures of functional status or disability (eg, symptoms, frailty metrics) should be presented.

### STEPS OF DATA PREPROCESSING.

The overarching goal of data preprocessing is to convert data to a format that allows AI algorithms to learn relevant information. For example, cardiovascular imaging data have large variability, including but not limited to videos with different cross-sectional views, frame rates, length, and scaling (ie, cm/pixel), along with differences in image acquisition procedures. In most cases, the selection of relevant view classes is included among the preprocessing steps to reduce variability in the input data,^27,28^ even though there are some exceptions for approaches using extremely large models.^29^ Which information should be made learnable by the model depends on the task the model needs to perform. For a task that only needs to calculate the ratio of the largest and smallest chamber size (ie, the automated calculation of ejection fraction), the absolute scaling and the frame rate may not be necessary.^30^ In contrast, an approach standardizing and/or passing information about the scaling and frame rate to the model may be more important when performing tasks where the relevant features are not entirely clear.^31^ This can be achieved by data harmonization (standardizing the frame rate of all videos to a specific value) or directly passing the information to the model. The data harmonization procedure and the data supplied to the model should be reported clearly.

Another important aspect of data preprocessing is to ensure the model does not learn irrelevant features. The specific challenge arises from the model’s ability to automatically extract features from complex, high-dimensional data sets. In theory, the model could use any features statistically associated with the label, including those certainly irrelevant to the task being achieved (eg, patient metadata text outside the imaging area,^32^ the vendor of the echocardiogram machine). It is critical that the authors report the procedure in the data preprocessing step that ensures the prevention of learning of these irrelevant features by removing them or testing that the model is not affected by the presence of the feature by performing subgroup analysis within those with the same feature (eg, subgroups that are imaged with a device from a specific vendor). Examples of the most common challenges that should be addressed during preprocessing are provided in Supplemental Table 3.

### FEATURE ENGINEERING AND FEATURE SELECTION.

Feature engineering converts raw data into representative inputs that improve model performance, whereas feature selection isolates the most relevant variables to minimize overfitting and unnecessary computational complexity.^33^ In cardiovascular imaging, common steps include normalization, encoding of categorical variables, and generation of composite indices such as ratios of volumetric metrics. Advanced approaches leverage DL-based representations or multimodal fusion of image- and ECG-derived features. Feature selection methods can be grouped into 3 categories: filter, wrapper, and embedded methods—where filter methods rank features via univariable tests (ideal for wide data sets), wrapper methods iteratively evaluate feature subsets with a model (best for small-to-medium data sets), and embedded methods integrate selection into training through regularization or tree-based importance.^34^ Nevertheless, feature selection is inherently incorporated in the training of DL models; thus, regularization is not always necessary.

In modern AI systems handling multimodal data and generative tasks, effective feature engineering has evolved into strategic representation design.^35,36^ Rather than manual feature crafting, researchers should focus on selecting appropriate pre-trained encoders (convolutional neural networks for images, transformers for text, variational autoencoders for structured data) that automatically extract hierarchical embeddings. For multimodal integration, critical steps include normalizing feature spaces through layer-specific norms and aligning modalities via contrastive learning or cross-attention mechanisms, ensuring balanced representation without modality dominance.^37^ Generative models particularly benefit from controlled latent spaces achieved through sparsity constraints, mutual information regularization, and domain-informed priors such as semantic tokenizers.^38,39^ While deep networks automate feature learning, incorporating structured inductive biases through pre-trained embeddings and implementing attention-based or gradient-driven dimension pruning significantly boosts generalization and efficiency. The fusion approach should be task-optimized, with cross-modal attention proving particularly effective for creating cohesive representations from early, intermediate, or late fusion strategies. This comprehensive approach simultaneously optimizes generative quality, multimodal coherence, and computational efficiency in state-of-the-art DL pipelines. The commonly used features engineering and selection techniques are presented in Table 4.

## SELECTION OF AI METHODS AND APPLICATIONS

### IMPORTANCE OF SELECTING APPROPRIATE AI METHODS AND APPLICATIONS.

Successful integration of AI within cardiovascular imaging requires selecting methodologies that align with clinical goals, data availability, and practice preferences. Choosing the appropriate method (ie, ranging from classical ML approaches to complex architectures such as transformers) is essential for optimizing performance while ensuring clinical relevance. Cardiovascular imaging workflows generate and use both structured (eg, images, signals) and unstructured data (eg, clinical notes),^40^ requiring careful selection and application of AI methodologies. In this context, model selection should balance interpretability and performance, as outlined in Table 5.

### TRAINING STRATEGIES FOR ML, DL, FOUNDATION, AND MULTIMODAL MODELS.

Broadly speaking, classical ML describes computer algorithms that learn to fit the training data provided. ML includes statistical algorithms (random forests, support vector machines, and more) as well as neural network-based algorithms, known as DL. These algorithms can be used to perform different types of tasks, such as classification, sequence prediction, or regression, on one or more of the data types mentioned earlier. As ML algorithms have become more sophisticated, larger, and more powerful, they have been increasingly used for multimodal tasks both within and outside medicine. This includes pairing image analysis with natural language processing (NLP) of associated text. For additional background on classical ML techniques, we refer the reader to our prior edition of the PRIME checklist.^1^

### EVOLUTION OF NEURAL NETWORK–BASED DL ALGORITHMS.

Neural network algorithms greatly advanced the application of ML to cardiovascular imaging because of their ability to extract image features and other spatial context for complex imaging data. In cardiovascular imaging, DL has been instrumental in advancing the automation and accuracy of diagnostic tools—from echocardiogram interpretation^27,41,42^ and coronary calcium scoring^43,44^ to myocardial tissue segmentation,^45,46^ screening,^47,48^ and risk stratification.^49^ Integration of DL models has evolved in cardiovascular imaging, predominantly beginning with analysis of static images^50^ to now capturing dynamic time-series modeling, image generation, and multimodal analysis.^51,52^ Important DL methods have included convolutional neural networks (CNNs), recurrent neural networks, generative adversarial networks (GANs), and vision transformers (Panel 1).

### FOUNDATION MODELS AND MULTIMODAL MODELS.

LLMs and large vision-language models (LVLMs) are increasingly being adopted across medical imaging thanks to their ability to encode and decode complex clinical semantics. LLMs describe DL models trained on vast corpora of text that are able to understand and generate human language, whereas LVLMs extend this capability by jointly learning from both text and image inputs.^53^ Both represent a powerful way to initialize models when dealing with limited data sets by enabling efficient fine-tuning and supporting their downstream generalizability. These models can serve as automated report generators, phenotype encoders, or natural language interfaces that connect clinical narratives with imaging data.^54^ In cardiovascular imaging, their multimodal capacity directly reflects how clinicians integrate information across different data types. Another transformative application of LLMs and LVLMs is the end-to-end automation of cardiovascular imaging reports through text decoders that convert the embeddings generated by trained image encoders into human-interpretable text (Supplemental Table 4, Panel 2).

### SOLVING CLINICAL PROBLEMS IN CARDIOVASCULAR IMAGING.

AI methods can be tailored to address specific clinical data types, ranging from structured tabular EHRs to complex imaging modalities. Understanding how different model families perform on these data types is essential for applying the appropriate AI tools to the given clinical problem. This section highlights appropriate algorithmic choices based on input data characteristics for cardiovascular imaging tasks.

#### Handling structured data.

Tabular data refers to structured data commonly found in EHRs, such as laboratory results, vital signs, demographics, medication lists, procedural codes, and data derived from imaging. These data sets are well-suited to classical ML models and increasingly leveraged for disease prediction,^55^ risk stratification, and health outcomes modeling^56^ in cardiovascular imaging. We will focus on choosing DL methods for handling structured data, with the original PRIME checklist^1^ as a reference for classical ML methods. When selecting DL models for cardiovascular imaging-derived tabular data, it is crucial to account for variability inherent to this domain, including nonstandardized acquisition protocols, data imbalance (eg, rare cardiac conditions or underrepresented populations), and inconsistent metadata across vendors,^57^ which can introduce significant noise and heterogeneity into the data set (Panel 3).

#### Image segmentation.

Imaging data, including echocardiograms, MRIs, computed tomography (CT) angiography, and nuclear perfusion scans, are rich in spatial information. They frequently involve dynamic structures with high beat-to-beat variability and motion artifacts caused by both cardiac and respiratory cycles. Additional important areas of variability include acquisition parameters (eg, probe positioning, contrast timing), inconsistent Digital Imaging and Communication in Medicine (DICOM) formats, artifacts introduced by cardiac support devices, and suboptimal gating. These complexities necessitate segmentation models that are not only accurate but also robust to temporal and spatial in-consistencies. Prominent DL architectures for image segmentation include CNN-based methods, importantly U-net and nnU-net, and vision transformers, such as the Swin Transformer. When creating synthetic data and augmenting training data, GANs, variational encoders, and diffusion models are the predominant methods (Panel 4).

#### Image-Based Reports.

Cardiovascular imaging reports often contain modality-specific terminology (eg, late gadolinium enhancement, coronary calcification), structured measurements, and nuanced interpretations of dynamic phenomena, all of which require algorithms to be trained or fine-tuned on medical data. Subjectivity in report phrasing poses additional challenges that can affect model consistency and accuracy. NLP models can be considered for curating and searching large databases, such as an EHR containing all transthoracic echocardiographic reports,^58^ or for generically classifying reports into normal vs abnormal stress echocardiograms^59^ (Panel 5).

#### Combining data sources.

Models trained on multi-source data outperform single-modality approaches, especially in complex clinical scenarios such as major adverse cardiac events.^60^ Some of the more prominent publicly available models that include both an LLM and a vision model include GPT-4V (GPT-4 with Vision).^61^ Ultimately, clinicians should decide whether to use a fine-tuned model (ie, specifically designed to learn aligned visual-text embeddings from a single imaging modality) vs a generalized model; there are tradeoffs between flexibility and required domain-specific fine-tuning (Panel 5).

### ADDITIONAL CLINICAL AI APPLICATIONS.

Beyond core AI model architectures, several adjacent technologies are shaping how AI is operationalized in cardiovascular imaging, including federated learning,^62^ edge learning,^63^ neuro-symbolic AI,^64^ and robotic process automation^65^ (Table 6).

## MODEL ASSESSMENT

### IMPORTANCE OF MODEL ASSESSMENT.

Model assessment measures a model’s performance on a specific data set using predefined metrics, whereas model evaluation encompasses broader judgments including generalizability, fairness, and clinical utility across diverse settings. A comprehensive evaluation framework must incorporate both statistical rigor and clinical relevance, considering data set imbalances, input modality (eg, imaging, EHR, text), and intended use. For AI adoption in cardiovascular imaging, such as analyzing cardiac MRI or summarizing echocardiographic findings, performance metrics must reflect both accuracy and reliability across diverse patient populations and care settings. A multilayered evaluation strategy supports regulatory approval, clinical trust, and successful integration into clinical workflows (Figure 1).

### TECHNICAL PERFORMANCE METRICS.

Technical performance metrics provide objective benchmarks for evaluating AI model accuracy, discrimination, and/or error distribution (Table 7).^30,66-77^ These metrics guide model selection, comparison, and tuning across diverse clinical applications. Selecting the appropriate metric depends on task type (classification, regression, segmentation, generation), data set characteristics (class imbalance, noise), and the clinical consequences of false positives or false negatives. Importantly, validation metrics are limited by the reference standard assessments that can have both a high intraobserver and interobserver variability^78,79^ in cardiovascular imaging. Table 8 provides pitfalls and considerations for common validation metrics across classification,^71^ segmentation,^80^ language,^81^ and multimodal tasks^82^ (Panel 6). For a more detailed description, please refer to the review by Maier-Hein et al.^72^

### ROBUSTNESS AND GENERALIZABILITY.

A model must not only perform well on internal validation but also demonstrate consistent accuracy when exposed to new patient populations, institutions, devices, and data quality variations. These characteristics ensure the safety, fairness, and equity of AI systems deployed across diverse clinical environments. For cardiologists, robust models translate to dependable diagnostics, prognostication, and workflow assistance, even in the face of variations in imaging protocols, documentation style, or patient demographics.

### EVALUATING DATA QUALITY.

Cross-validation helps estimate the performance of a model by splitting the data into multiple subsets for iterative training and validation. Internal cross-validation may still over-estimate performance due to hidden correlations or site-specific imaging features that do not generalize to external data sets. Additionally, because some segmentation tasks suffer from higher interobserver variability, such as identifying high-risk plaque morphology,^83^ cross-validation might not fully capture the impact of label subjectivity. External validation evaluates model performance on entirely independent data sets not used during training. The use of federated learning, as discussed above, can act as a potential solution for sharing robust, annotated cardiovascular imaging data sets between institutions.^84^ Even robust models may suffer due to nonstandardized imaging protocols (eg, MRI sequence differences or CT contrast injection timing) that are hard to harmonize across institutions. Ultimately, robust models must perform consistently across a wide range of data characteristics. Differences in imaging quality, scanner protocols, EHR completeness, patients’ demographics, or variations in input data, such as changes in noise or brightness, can degrade model accuracy (Panel 7).

### IDENTIFYING FEATURES LEARNED BY THE MODEL.

#### AI model interpretability, explainability, and bias/fairness.

AI tools, and in particular DL models, are still considered black boxes that assign a diagnostic category after complex operations on the input data. A major challenge for medical applications is to better understand such decisions by targeting interpretability and explainability, which are encompassed under the umbrella of transparency^58^ and, to a broader extent, trustworthiness.^85^ Interpretability refers to understanding how the AI model led to a given classify an individual subject, whereas explainability refers to explaining the final decision made and therefore requires a lower level of detail. Transparency can be tackled post hoc or be intrinsic to the model. Some widespread post hoc methods include scores that grade feature importance to the decision,^86^ and attribution maps at the scale of image pixels or text elements.^87,88^ At stake is the insurance that the AI tools provide equitable care across patients (eg, of diverse age, sex, race, and socioeconomic groups), and that decisions are clinically relevant (eg, regarding physiological traits of the disease, structures of interest in the analyzed images, consistent decisions for comparable patients or risk groups, and so on).

General recommendations start with identifying and quantifying the bias in the decision^89^ to ensure fairness^90^: similar prediction rates across groups (demographic parity) and, to a finer extent, true positive rates across groups (equal opportunity), and true positive and false positive rates across groups (equalized odds). The mitigation of such biases can be addressed before, during, and after the training phase^90^ by respectively operating on the data distribution, model robustness, and post-processing of the model outcomes, with implications beyond technical performance of the model up to ethical considerations for the patient and the health care system (Panel 8).

#### Uncertainty.

Medical data analysis is prone to un-certainties in the decision at 4 different stages: data collection, data labelling, model selection, and model-based inference.^91^ The types of uncertainty consist of aleatoric and epistemic uncertainty, which respectively correspond to how much the output decision is affected by variations in the input data, or by a lack of knowledge on such data (namely, model uncertainty). Although the latter is more covered in the published reports, the value of uncertainty quantification and communication is often under-estimated while it could play a major role for health care applications^92^ (Panel 9).

#### Clinician acceptance and iterative refinement.

Successful clinical adoption of AI models depends on user trust, which requires both technical rigor and effective communication. These include transparent presentation of model uncertainty in clinically interpretable formats (eg, CIs, risk stratification thresholds), use of post hoc interpretability tools (eg, Shapley additive explanations values, saliency maps, attention visualizations), and structured opportunities for clinician-in-the-loop feedback. Iterative refinement strategies, such as targeted error review sessions, model-assisted annotation workflows, and retraining informed by clinician feedback, should be documented to demonstrate alignment between model outputs and expert judgment. Incorporating these practices fosters transparency, mitigates over-reliance on deterministic outputs, and facilitates sustainable integration of AI tools.

## CLINICAL EVALUATION

### IMPORTANCE OF CLINICAL EVALUATION.

AI models should first be evaluated using statistical metrics on comprehensive and diverse data sets. Test data should be independent of the training data collected from a different population and at different clinical sites. A test data set should also have a sufficient sample size^67^ and be representative of the population that it will be applied to across age ranges, sex, ethnicity, and other patient characteristics. Model performance should be compared between patient groups to address any biases and ensure equality and generalizability.

AI model performance measured by statistical methods may not directly translate to clinical utility and disease-specific contexts. For example, diagnostic images that have statistical similarities may differ in diagnostic values and suggestions. Misclassification in AI methods should be weighted by the consequences of misdiagnosis in clinical settings. In this sense, model testing in clinical AI should extend beyond metrics, to include evaluation by medical experts in real-world clinical workflow (Table 9, Figure 2).^93,94^

### CLINICAL UTILITY METRICS.

Performance of an AI tool should be interpreted in clinical contexts. For example, errors in automated image analysis and quantification should be assessed by their downstream impact on disease classification, grading, and therapeutic decisions. Assessment of a new AI diagnostic solution with reported metrics such as accuracy, sensitivity, specificity, positive predictive value, negative predictive value, should be put in context of clinical consequences^72^: false positive and misdiagnosis may lead to unnecessary procedures or anxiety incurring additional cost and invasiveness; false negatives could delay treatment leading to high cost in subsequent patient care. Depending on the settings and purpose of the medical test, an AI tool may prioritize sensitivity (eg, for screening) or specificity (eg, for gating or triage). Specific attention should be paid to biases and misdiagnoses affecting underrepresented groups, due to the tendency of AI to fit predominantly to prevalent characteristics.

To assess clinical decision support tools, decision curve analysis (DCA) and net clinical benefit (NCB) are established frameworks that are also applicable to AI-based methods. DCA evaluates the net benefit of using a model across decision thresholds by considering the harms of false positives and negatives. NCB quantifies the clinical value of model-assisted decisions over standard care by incorporating the benefits and costs of correct and incorrect interventions. These tools help determine whether an AI model translates to actual improvements in patient care. Additionally, clinical value should be supported by economic viability (Panel 10).

### CLINICAL VALIDATION.

The clinical utility of AI systems can be assessed across multiple domains, including patient outcomes (eg, mortality, incidence of major cardiovascular events), clinical efficiency (eg, time per case, time to clinical decision), diagnostic accuracy (eg, rate of correct diagnoses, clinician diagnostic confidence), and economic impact (eg, cost-effectiveness, reduction in health care resource use).

The extent of clinical validation should be proportionate to the potential risk of the AI system, which is influenced by the extent of human oversight and the intended clinical use of the AI.^95^ The IMDRF (International Medical Device Regulators Forum) proposed a structured risk categorization framework that depends on the clinical context for which the AI is intended and the significance of the information provided by the AI.^96^

RCTs provide the strongest clinical evidence, and they may be necessary before clinical decision support software can be implemented safely. In lower-risk systems, real-world evidence can offer an alternative to formal trials, where AI performance is measured from observational data captured from routine clinical practice. Real-world evidence can capture a representative patient population, including noncurated data with missing values, and reflects real-world variables such as clinician variability, workflow integration, and system interoperability. Validating on such real-world data can assess the generalizability and external validity of an AI system. In silico trials offer a complementary approach, enabling simulation-based validation across virtual patient cohorts before or alongside real-world deployment.^97^

### CONTINUOUS MONITORING.

AI model performance can decay over time due to changes in data (eg, change in patient demographics), image acquisition (eg, new scanner, change of imaging protocol), or clinical practice (eg, an updated disease definition). Failure to detect model performance degradation, even in small population subgroups, can lead to biased predictions, safety risks, and reduced trust. Model performance should be continuously monitored and evaluation metrics periodically collected to identify the need for model refinement. Thresholds for triggering model refinement should be predefined and be clinically relevant. If performance degradation is detected, the precise cause should first be sought and understood to help guide the approach to model refinement (eg, by updating training data [retraining], model parameter values [recalibrating], or improving the modelling structure or training method). When a model is refined, all tests performed before the initial model deployment should be re-evaluated to ensure no detriment in performance.

A plan for model and software maintenance should be established at the time of model deployment, which can avoid the need to submit a new application for regulatory approval whenever a new model is used. The U.S. Food and Drug Administration, eg, refers to this as a predetermined change control plan.^98^ As part of this process, all modifications should be described, along with the modification protocol (the retraining process, performance evaluation, data management, and the update mechanism), as well as impact assessment (effect on performance, risk analysis, and risk mitigation). Further discussion of post-market surveillance and post-deployment management as it pertains to regulatory bodies will be covered in an accompanying paper.

AI model monitoring, retraining systems, and regulatory considerations are all considered as part of a wider emerging field called ML operations, which aims to provide a process to support AI tools after deployment.^99^

## BEST PRACTICES FOR REPLICABILITY

### IMPORTANCE OF TRANSPARENCY AND OPEN SCIENCE PRINCIPLES.

Transparency and open science align with the FAIR (findable, accessible, interoperable, and reusable) principles of scientific data (Supplemental Table 5)^100^ and are key for collaborative innovation. Transparency is recommended to allow scrutiny and reproducibility in AI research and to ensure that developed tools are accessible and trustworthy and provide unbiased benefits across different population groups. Thus, transparency about the underlying training data and ensuring that these are representative across populations is recommended.

An equitable open-source model for data sharing is recommended whenever possible to promote scientific discovery, reproducible research, and clinical translation.

### ENSURING TECHNICAL REPRODUCIBILITY.

Technical reproducibility of the results (ie, the ability to obtain the same results with the same methodology and data) is an essential part of scientific reporting. This process can be approached hierarchically—at the level of the model, the overall methodology, and the entire study. However, applying DL to complex data presents unique challenges to this approach.

One considerable challenge for technical reproducibility unique to the medical field is the difficulty of publishing the clinical data set. Clinical data may contain patient-identifiable information. In DL, this also translates to the difficulty of publishing the model weights, as large models can be vulnerable to privacy attacks capable of extracting sensitive data from training inputs.^101^ Although sharing data sets and model weights is encouraged to enhance reproducibility, it is not always feasible. Additionally, intellectual property and commercialization issues limit data and model sharing. In such cases, a clear justification for any restrictions should be provided.

Another major challenge is the difficulty of replicating the training process. Even when using the same model architecture, data set, and hyper-parameters, the resulting model may differ due to inherent sources of randomness. For example, stochastic operations on graphical processing units can introduce variability that is difficult or impossible to eliminate completely.^102^ Therefore, it is essential to clarify the intended target of reproducibility when reporting results. In most cases, the goal is either to reproduce the final trained model or to replicate the entire training methodology. The materials and documentation required for reproducibility depend on this target and should be reported accordingly.

When reporting only the final model, randomness does not have a significant effect as the model parameters are fixed, and the same input is expected to yield consistent output. In this context, reproducibility can be achieved by providing model architecture, trained weights, and the test data set used for evaluation. These materials can be shared as source code, model weight files, and data sets through publicly accessible repositories (Supplemental Table 6). However, as noted earlier, publishing certain model weights or data sets may not be feasible due to privacy concerns or data-sharing restrictions. In such cases, a clear justification for the unavailability of these materials should be provided.

When reporting the entire methodology for producing a model, it is essential to account for all sources of randomness that may affect the training process. These include factors such as weight initialization, random sampling of the data set, and stochastic components of the training procedure (eg, dropout layers, learning rate schedules, and reduction methods), as well as hardware-specific behaviors. To assess the robustness of the methodology, the statistical variability should be quantified by repeating the training process multiple times and reporting the resulting performance distribution. In this context, authors should provide the source code encompassing the full training pipeline, along with details such as random seed values, hardware specifications, and all materials typically required for final model reporting.

### SPECIFIC CONSIDERATIONS FOR REPRODUCING LLM AND GENERATIVE AI STUDIES.

Reproducing studies involving LLMs and generative AI requires careful attention to the following factors. Many state-of-the-art models (eg, GPT-4) are proprietary, and pre-training data is often unavailable or undocumented. Because these models are frequently updated and their internal details are often undisclosed, authors should report the exact model name, version, provider, and date of access. Prompt details must also include the exact wording, use of templates or few-shot examples, and generation parameters such as temperature or max tokens. The context of model use should be described, including application programming interface version and call timing for cloud-based models, or base model and fine-tuning procedures for local models.

Given the stochastic nature of LLM outputs, researchers should clarify how output variability was handled, eg, by reporting multiple runs, random seeds, or summary statistics. Where full disclosure is not possible, the possibility for independent testing and benchmarking should ideally be offered.

## REPORTING LIMITATIONS, BIASES, AND ALTERNATIVES

### ACKNOWLEDGING STUDY AND MODEL LIMITATIONS.

Accurate reporting and acknowledgment of limitations are critical to any scientific research. In studies involving AI methodologies, such limitations may arise from multiple aspects. Table 10 presents major categories of limitations, with examples and assessment methods. These include data-related limitations such as limited scope or representativeness (eg, small sample sizes, single-center design, limited diversity), quality concerns (eg, missing data, noise, artifacts), and labeling challenges (eg, interobserver variability, lack of ground truth). Methodological limitations involve model assumptions, architectural or optimizer constraints, and simplifications such as resolution down-sampling. Generalizability concerns relate to population, technical, and clinical contextual differences, and should be addressed by reporting subgroup performance or degradation in external settings. Sensitivity analyses are encouraged to assess robustness across variations in data, parameters, and prompts, especially for advanced models. Finally, model-specific limitations, such as hallucinations in LLMs, controllability in generative AI, and challenges in interpretability, should be transparently discussed to support responsible clinical use.

### DISCUSSING STUDY STRENGTHS.

Methodological rigor should be discussed, emphasizing data harmonization, robust cross-validation, and systematic benchmarking across unimodal and multimodal baselines. Key innovations include the integration of emerging architectures, such as multimodal AI and LLMs, into traditional clinical pipelines, enhancing both predictive performance and interpretability. Strengths related to data set characteristics, including real-world diversity, longitudinal follow-up, and class balance, support generalizability across clinical scenarios. Importantly, the checklist framework aligns with established diagnostic workflows and prioritizes clinical relevance through model explainability, calibration, and actionable outputs. By articulating these strengths clearly, studies can highlight both their technical contributions and translational readiness, facilitating rigorous evaluation and meaningful clinical impact in cardiovascular AI research.

### REPORTING ON BIAS AND FAIRNESS ASSESSMENT.

Studies must systematically identify, assess, and report potential biases affecting model development through comprehensive fairness evaluation frame-works. Key biases include selection bias (eg, differences in development cohorts due to referral patterns or access to care), representation bias (eg, lack of demographic diversity or exclusion of sub-groups), measurement bias (eg, variations in data acquisition protocols or image quality across patient groups), annotation bias (eg, differences in expert interpretation by institution or patient characteristics), historical bias (eg, embedded disparities in legacy data), and algorithmic bias (eg, model architectures that favor certain populations). These bias types are summarized in Table 11.

Fairness evaluation should include quantitative metrics with performance stratified by demographic subgroups. Statistical testing with CIs and effect sizes should identify disparities, particularly those with clinical implications. Where possible, intersectional analyses should be included. In addition, bias mitigation strategies should be reported in detail, including pre-processing (eg, data augmentation, resampling), in-processing (eg, fairness-aware training, adversarial debiasing), and post-processing (eg, threshold optimization, calibration correction). Authors must discuss the effectiveness and limitations of these approaches, including fairness-performance trade-offs and the risk of unintended consequences.

Residual bias analysis should assess the remaining biases and their potential clinical impact, particularly for historically marginalized populations. Studies should consider whether AI models might perpetuate or exacerbate existing inequities.

Finally, limitations in the fairness assessment it-self must be acknowledged. These may include data availability, small subgroup sizes, limited generalizability to real-world deployment, and uncertainty regarding long-term fairness in evolving health care systems.

### CONTEXTUALIZING WITH ALTERNATIVE METHODS.

To appropriately contextualize AI models, studies should benchmark their performance against clinical standards, traditional risk scores, and simpler statistical models. For example, AI-based ejection fraction estimation has demonstrated strong correlation with the gold-standard biplane Simpson’s method,^103-105^ and DL models trained on more than 300,000 echocardiograms have outperformed logistic regression in survival prediction tasks.^106^ Studies have shown that advanced architectures, such as CNNs and LLMs, have matched or surpassed expert performance in tasks including view classification, arrhythmia detection, and coronary disease assessment.^107-111^ Such comparisons provide a foundation for evaluating the added value of advanced AI techniques.

The use of complex architecture, such as DLs and LLMs, should be explicitly justified by superior performance. Studies have shown that these models can match or exceed expert-level accuracy in view classification, chamber segmentation, and disease detection, often achieving areas under the curve >0.90.^93,112^ Multimodal models have also outperformed traditional risk scores in event prediction, and systematic reviews support their potential to improve image quality and diagnostic consistency.^113^ These findings reinforce the rationale for adopting more sophisticated AI models in cardiovascular imaging.

Although AI models often outperform conventional methods, non-AI computational approaches continue to play an important complementary role in contextualizing and validating AI outputs. Techniques such as radiomics, topological data analysis, and physiological simulations can enhance interpretability, provide benchmark references, and align predictions with clinical reasoning. Radiomics enables quantitative feature extraction from imaging data, supporting risk stratification and comparison with AI-based pre-dictions.^114^ Topological data analysis captures complex geometric and structural patterns that may be missed by conventional pixel-level analyses.^115,116^ Physiological models, including simulations of strain or blood flow, offer mechanistic validation by grounding AI outputs in established biological principles.^117^ The integration of such methods supports transparency, improves model credibility, and facilitates alignment with clinical expectations.

## CONCLUSIONS

PRIME 2.0 addresses the pressing need for structured, transparent, and reproducible reporting of AI studies in cardiovascular imaging. It offers detailed, domain-specific recommendations that reflect both the complexity of imaging data and the rapid advancement of AI methods, ensuring that research meets the expectations of scientific rigor and clinical relevance. To uphold these standards across published reports, a completed PRIME 2.0 checklist can be submitted as supplemental material with the research manuscript to support transparency and enable peer reviewers and readers to critically assess methodological robustness.

## Supplementary Material

Extended Checklist

Supplementary Table 7

Supplementary tables and references

## Figures and Tables

**FIGURE 1:**
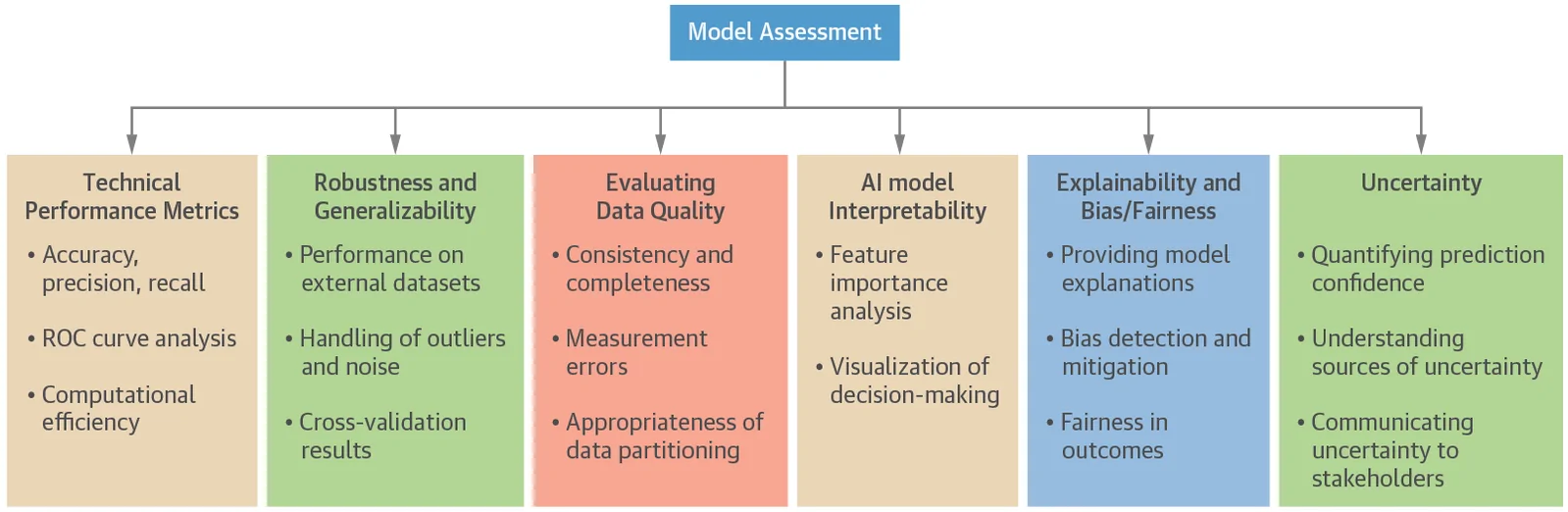
Factors to Be Assessed When Evaluating an AI Model Six critical domains for evaluating artificial intelligence (AI) models: technical performance metrics, robustness and generalizability, data quality, model interpretability, bias/fairness, and uncertainty. Together, these domains provide a structured framework to ensure transparent, reliable, and clinically applicable AI model validation.

**FIGURE 2:**
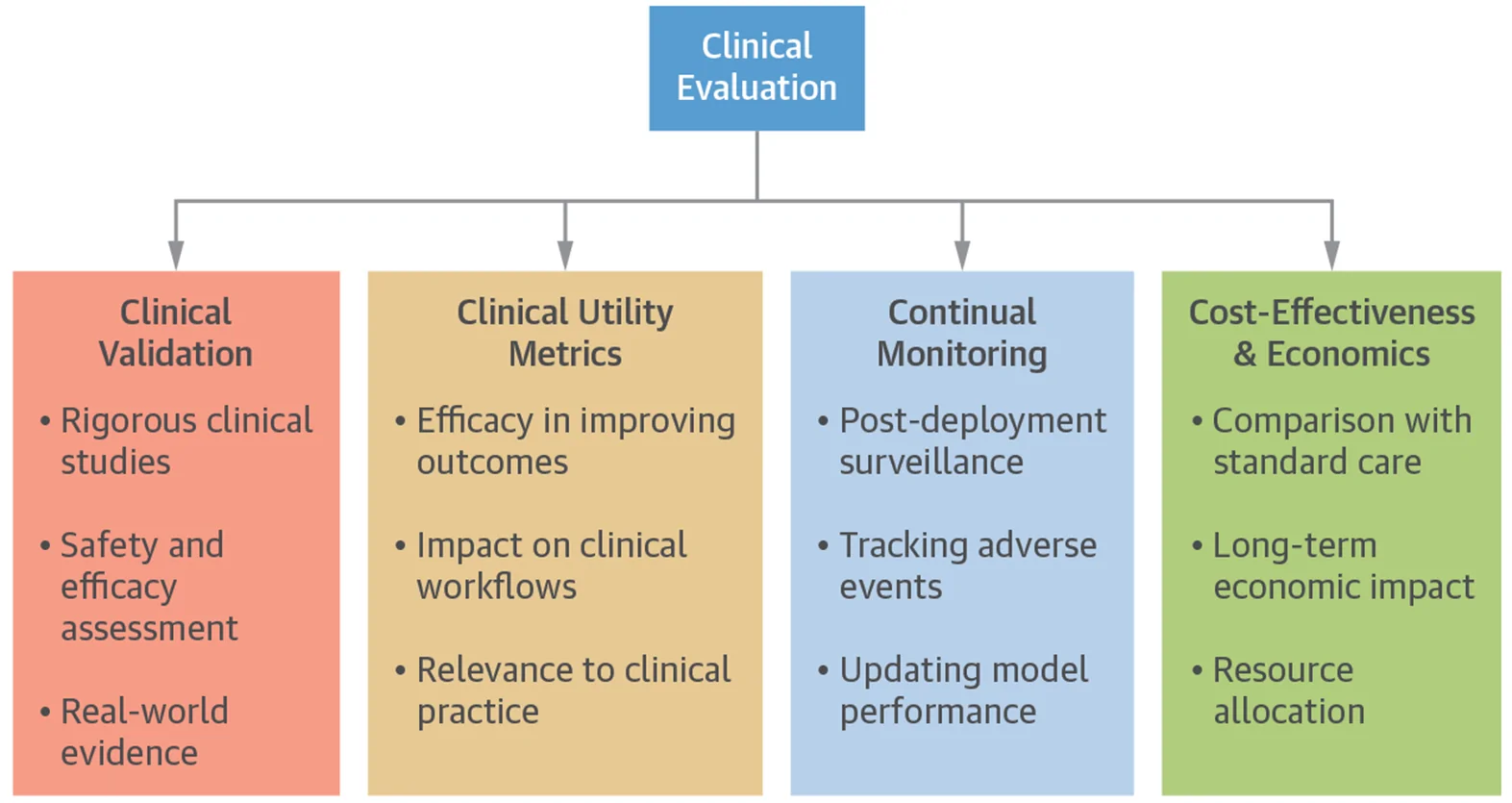
Priorities for Clinical Evaluation of AI Models Four key priorities in evaluating AI models for clinical use: clinical validation, utility metrics, continual monitoring, and cost-effectiveness. These dimensions help ensure AI solutions are safe, effective, relevant to workflows, and economically viable in real-world health care settings. Abbreviation as in Figure 1.

**TABLE 1:** Challenges for Using AI in Cardiovascular Imaging Research

Common challenges	
Dynamic heart motion	Analysis of dynamic sequences or videos requiring advanced temporal modeling to accurately capture cardiac function
ECG gating	Challenges in accurately synchronizing image acquisition with specific phases of the cardiac cycle
High anatomical variability	Diverse anatomies across different patients necessitating adaptable and robust AI models
Subjective reference standards	Difficulty in establishing consistent ground truth due to high interobserver and intraobserver variability
Standardization of imaging protocols	Variations in acquisition protocols across centers affecting reproducibility and comparability
Image quality variability	Challenges in consistently maintaining and assessing image quality due to patient movement, operator skill, and equipment variations
Data imbalance	Rare cardiovascular events or diseases and underrepresented patient subgroups causing training biases and limited model performance
Echocardiography	
Acquisition variability	Significant variations in field-of-view, gain, imaging depth, and probe positioning affecting image quality
Inconsistent DICOM metadata	Metadata inconsistency across ultrasound vendors complicating data harmonization and sharing
Ultrasound artifacts	Frequent artifacts such as reverberation, shadowing, attenuation, and speckle noise impacting image interpretation
Complex Doppler integration	Difficulties in the analysis and interpretation of spectral Doppler, color Doppler, and tissue Doppler signals
CT and MRI	
Contrast injection timing	Variability in contrast timing influencing image clarity, contrast enhancement, and consistency of anatomical visualization
Protocol variability	Differences in imaging protocols, particularly in MRI sequences, complicating standardization and reproducibility
Imaging artifacts Angiography	Distortion or signal degradation from cardiac motion, respiratory movements, metal implants, or patient-specific conditions
Angiography	
Imaging angle variability	Significant differences in imaging angles and projections affecting the consistent visualization of coronary structures
Motion-induced artifacts	Image distortion due to cardiac and respiratory motion during the acquisition process
Contrast injection variability	Variations in contrast agent concentration, volume, and injection speed impacting diagnostic accuracy
Nuclear imaging	
Spatial resolution, noise	Limitations in spatial resolution and high background noise complicating precise anatomical and functional interpretations
Radiotracer variability	Differences in radiotracer types and imaging protocols affecting image comparability and reproducibility
ECG and signal data	
Ambiguous data representation	Challenges in clearly distinguishing between ECG signals and derived image representations, impacting model training and interpretation
Nonstandardized formats	Variability in storage formats complicating data integration, analysis, and sharing
Signal acquisition variability	Differences in lead configurations, sampling rates, and filtering methods impacting consistency and model accuracy

AI = artificial intelligence; CT = computed tomography; DICOM = Digital Imaging and Communications in Medicine; ECG = electrocardiogram; MRI = magnetic resonance imaging.

**TABLE 2:** The Checklist for Standardized Reporting With Essential and Optional Items

Section	Checklist #	Topic	Checklist Item
1. Designing an AI Study in Cardiovascular Imaging	1.1	■ Appropriateness of applying AI	Describe the need for applying AI Determine the appropriateness of applying AI
	1.2	■ Study objectives, input data type, and prediction target	Explain the AI task and the likely deployment context Describe the input data, number of training/test examples Specify model supervision type Describe the nature of the model’s output and what it represents
	1.3	■ Design of the AI study	Describe the study design Describe data origin Describe if impact analysis was included
2. Data Format and Preprocessing	2.1	■ Data format	Describe the technical details of the data acquisition Describe the technical details of the data format
	2.2	■ Clinical characteristics of the study cohort	Present the age, sex, and race/ethnicity distributions of the cohort(s) Summarize key clinical, treatment, and imaging characteristics of the cohort(s) Compare summary statistics of cases and controls
	2.3	■ Steps of data preprocessing	Describe how data were cleaned, made uniform, and consistent Describe data harmonization techniques (if applicable) Provide details on missing values and imputation methods Describe processes for handling outliers Describe whether class imbalance exists
	2.4	■ Feature engineering and feature selection	Describe applied feature engineering techniques Describe applied feature selection techniques
3. Selection of AI Methods and Applications	3.1	■ Selecting appropriate AI methods and applications	Clearly define data composition (structured/unstructured)
	3.2, 3.3, 3.4	■ Training strategies	Describe the AI method/application used, with rationale for clinical task fit
	3.5	■ Solving clinical problems	For segmentation tasks, describe architecture choice and temporal data handling For NLP/report generation, indicate domain-specific fine-tuning and evaluation If combining multiple data sources, provide rationale and integration method
4. Model Assessment	4.1	■ Importance of model assessment	Describe the evaluation approach and how it addresses the clinical question
	4.2	■ Technical performance metrics	Report relevant performance metrics (F1-score, IoU, BLEU, etc) Justify metric selection based on task characteristics Describe manual annotation process, reference-standard prep, and observer variability
	4.3, 4.4	■ Robustness, generalizability, and evaluating data quality	Evaluate model robustness to external variations (scanners, protocols, institutions) Assess performance across clinical subpopulations and sociodemographic subgroups
	4.5	■ Identifying features learned by the model	Describe interpretability/explainability methods Quantify and discuss uncertainty (epistemic, aleatoric)
5. Clinical Evaluation	5.1	■ Importance of clinical evaluation	Describe potential clinical impact of misclassification
	5.2	■ Clinical utility metrics	Define clinical utility of the AI system Describe cost-effectiveness analysis (throughput, resource utilization)
	5.3	■ Clinical validation	Provide evidence of clinical validation
	5.4	■ Continuous monitoring	Outline plans for post-deployment monitoring
6. Best Practices for Replicability	6.1	■ Importance of transparency and open science principles	Ensure data sharing follows FAIR principles Report on training data representativeness
	6.2	■ Ensuring technical reproducibility	Report results for the final model and training process Describe sources of randomness in training Report random seeds (if applicable) Describe hardware setup Evaluate uncertainty from randomness (multiple training runs) Justify if source code, model weights, or data sets are not shared
	6.3	■ Specific considerations for reproducing LLM and generative AI studies	Report model name, version, provider, and access date Describe exact prompts and key generation parameters Clarify usage context (API vs local, fine-tuning) Explain approach to handling output variability Enable independent testing if full reproducibility is not possible
7. Reporting of Limitations, Biases, and Alternatives	7.1	■ Acknowledging study and model limitations	Discuss key limitations (data, methodology, generalizability) Report sensitivity analyses and model-specific issues
	7.2	■ Discussing study strengths	Articulate strengths (methodological rigor, data set quality, innovation, clinical relevance)
	7.3	■ Reporting on bias and fairness assessment	Report stratified performance metrics for demographic subgroups Describe bias mitigation strategies and residual bias Acknowledge fairness evaluation limitations
	7.4	■ Contextualizing with alternative methods	Benchmark against clinical standards/traditional risk scores Justify complex AI models over simpler alternatives Discuss complementary methods (eg, radiomics) for validation

The checklist template is provided for researchers in Supplemental Table 7.

■ = essential; ■ = optional; API = application programming interface; BLEU = Bilingual Evaluation Understudy (a metric for evaluating text generation); DL = deep learning; FAIR = findable, accessible, interoperable, reusable (principles for data management and stewardship); F1-score = harmonic mean of precision and recall (used for classification evaluation); IoU = intersection over union (used for segmentation accuracy evaluation); LLM = large language model; ML = machine learning; NLP = natural language processing; other abbreviation as in Table 1.

**TABLE 3:** Examples of AI Clinical Study Designs

	First Author, Study Name	Aim of the Study	Primary Endpoint	Single-Center/ Multicenter	N
Retrospective study	Knott et al^11^	To assess the prognostic value of myocardial blood flow and myocardial perfusion reserve by AI-based MRI perfusion mapping	Death or MACE	Multicenter	1,049
Prospective registry	Miller et al^12^	To assess the prognostic impact of deep learning-quantified body composition in PET/CT images	Death or myocardial infarction	Multicenter	10,085
Prospective study (single-arm)	Han et al^14^	To evaluate changes in deep learning-quantified coronary plaque composition on CT and PET after evolocumab treatment	Change in noncalcified coronary artery plaque volume	Single-center	47 (196 lesions)
Prospective registry, post hoc analysis of RCT	Lin et al^15^	To develop a deep learning system for plaque volume and stenosis assessment on CT and to assess its prognostic value	Agreement between deep learning and expert measurements, myocardial infarction	Multicenter	921 (5,045 lesions) for training, 275 (1,901 lesions) for testing, 1,611 for prognostic value evaluation
Prospective RCT	Upton et al,^16^ PROTEUS	To evaluate the noninferiority of AI-augmented vs standard clinical decision-making in stress echocardiography	Appropriate referral for coronary angiography	Multicenter	2,341
Pragmatic RCT	Petch et al,^17^ CarDIA-AI (design paper)	To evaluate the role of AI-based risk assessment in optimizing the use of invasive coronary angiography among outpatients referred for nonurgent evaluation	The proportion of normal or nonobstructive coronary stenosis diagnosed via invasive coronary angiography	Multicenter	252
Randomized crossover trial	Sakamoto et al,^21^ AI-ECHO RCT	To evaluate the impact of AI-based automated echocardiographic analysis on sonographer work efficiency	Workflow efficiency, defined as the number of daily examinations per sonographer and the time required per examination	Single-center	585 (38 days, day-by-day randomization)

AI-ECHO = Artificial Intelligence-based automated ECHOcardiographic measurements and the workflow of sonographers; CarDIA-AI = Coronary computed tomographic angiography to optimize the Diagnostic yield of Invasive Angiography for low-risk patients screened with Artificial Intelligence; MACE = major adverse cardiac events; PET = positron emission tomography; PROTEUS = a PROspective randomised controlled Trial Evaluating the Use of artificial intelligence in Stress echocardiography; RCT = randomized controlled trial; other abbreviation as in Table 1.

**TABLE 4:** Commonly Used Techniques for Feature Engineering and Selection

	Technique	Common Use Cases	Key Benefits	Examples
Basic feature engineering	Normalization/scaling	All data types	Stabilizes training, improves convergence	Min-Max scaling, Z-score transformation
	Categorical encoding	Tabular data	Converts non-numeric features	One-hot encoding, label encoding
	Composite feature creation	Domain-specific (eg, medical imaging)	Captures higher-order relationships	Volumetric ratios, temporal derivatives
Representation learning	Pretrained encoders	Modality-specific data	Automates feature extraction	CNNs (images), transformers (text), VAEs (structured)
	Contrastive learning	Multimodal alignment	Aligns cross-modal features	CLIP, triplet loss
	Cross-attention mechanisms	Multimodal fusion	Dynamically weights relevant features	Perceiver, Flamingo
Generative AI	Sparsity constraints	Latent space regularization	Improves interpretability	L1 regularization, top-k attention
	Mutual information regularization	Disentangled representations	Prevents feature collapse	InfoGAN, VAE variants
	Domain-informed priors	Task-specific guidance	Incorporates expert knowledge	Semantic tokenizers, anatomical atlases
Feature selection	Filter methods	High-dimensional data	Fast, model-agnostic	Correlation, ANOVA
	Wrapper methods	Small/medium data sets	Optimizes for model performance	Recursive feature elimination
	Embedded methods	End-to-end training	Balances selection and learning	LASSO, tree-based feature importance
Fusion strategies	Early fusion	Simple multimodal tasks	Low-complexity integration	Feature concatenation
	Intermediate fusion	Complex cross-modal interactions	Learns cross-modal relationships	Cross-attention layers
	Late fusion	Independent modality processing	Flexible, modular architecture	Ensemble averaging

ANOVA = analysis of variance; CLIP = contrastive language–image pretraining; CNN = convolutional neural network; InfoGAN = information maximizing generative adversarial network; LASSO = least absolute shrinkage and selection operator; VAE = variational autoencoder; other abbreviation as in Table 1.

**TABLE 5:** Guidance on Selecting AI Models Based on Interpretability and Performance Tradeoffs

Model Type	Typical Performance Characteristics	Interpretability (Transparency and Explainability)	Key Use Cases in CV Imaging	When to Prefer This Model
Classical ML (eg, logistic regression, random forests)	Moderate performance on structured/tabular data	High (feature importance easily understood)	Risk prediction, tabular EHR data	When transparency is critical (regulatory or early clinical deployment)
Conventional DL (CNN, RNN, U-net)	High accuracy for imaging and signal data	Moderate (post hoc explainability via saliency maps)	Image segmentation, cine MRI/echo analysis	When performance is prioritized but local feature attribution is still needed
Advanced DL (Transformers, attention-based CNNs)	Very high performance, robust to data variability	Low–moderate (attention maps offer limited interpretability)	Multimodal fusion, complex imaging pipelines	When accuracy on heterogeneous data sets is paramount, and limited interpretability is acceptable
Foundation/LLMs and LVLMs	High adaptability across tasks, strong generalization	Low (complex architectures; explainability via prompt-level interpretability or grounding)	Report summarization, multimodal tasks (echo + text)	When handling unstructured data or integrating multiple modalities
Hybrid or neuro-symbolic AI	High performance with built-in reasoning constraints	High (explicit rule-based components)	Bias detection, guideline-constrained decision support	When both interpretability and safety are non-negotiable
Generative models (GANs, diffusion)	High (data synthesis, augmentation)	Low (outputs evaluated rather than model internals)	Rare phenotype modeling, data augmentation	When improving downstream model performance or mitigating class imbalance

CV = cardiovascular; EHR = electronic health record; GAN = generative adversarial network; LVLM = large vision-language model; RNN = recurrent neural network; U-net = variant of convolutional neural networks designed for image segmentation; other abbreviations as in Tables 1 to 4.

**TABLE 6:** Adjacent Technologies Shaping AI Use in Cardiovascular Imaging

Emerging AI Applications	Description	Example
Federated learning	Enables decentralized training of machine learning models across multiple institutions or devices without transferring raw patient data.^62^ This approach preserves patient privacy and aligns with data governance policies such as HIPAA.	Training diagnostic models across international sites, improving generalizability while mitigating biases from single-center data sets.
Edge learning	Brings AI inference closer to the data source, enables real-time analysis on devices such as wearable ECG monitors, portable ultrasound scanners, or smart stethoscopes.^63^	AI-enabled stethoscopes can detect valve disease during routine exams without requiring internet connectivity, which can also alleviate privacy concerns.
Neuro-symbolic AI	Integrates data-driven neural networks with rule-based symbolic reasoning; this technique bridges the gap between the pattern-recognition capabilities of deep learning (eg, CNNs for image segmentation and feature extraction) and the structured domain knowledge encoded in symbolic representations such as ontologies and knowledge graphs.^64^	Neural networks can identify complex anatomical structures in cardiac MRIs, while symbolic rules can guide interpretation by linking imaging biomarkers (eg, myocardial scar volume, left ventricular mass) to clinical guidelines or pathophysiological models.
Robotic process automation	Manages repetitive administrative and clinical processes using rule-based bots, enhancing operational efficiency.^65^	Could streamline scheduling of follow-up visits for post-MI patients, generate structured reports from echo findings, and automate insurance authorizations for cardiac procedures.

HIPAA = Health Insurance Portability and Accountability Act; MI = myocardial infarction; other abbreviations as in Tables 1, 3, and 4.

**TABLE 7:** Recommendations for Interpretation of Performance Metrics

Sample size	Power Analysis based on AUC or accuracy α (alpha): Type I error rate (false positive), typically 5% Power (1-β): Probability of detecting a true effect, usually 80% to 90% Effect size: Expected difference or strength of association (eg, AUC) Variance: SD or expected variability of the measure
Intraobserver variability	Kappa statistics^66^ Almost perfect: >0.81 Substantial: 0.61 and 0.80 Moderate: 0.41 and 0.60 Fair: 0.21 and 0.40 Slight: 0.01 and 0.20 ICC^67^ Excellent: >0.90 Good: 0.75 and 0.90 Moderate: 0.50 and 0.75 Poor: <0.50 Bland-Altman^68^ 95% LoA should be clinically acceptable (defined by domain experts) A small bias with narrow LoA is ideal
Intraobserver variability	Coefficient of variation^69^ Excellent: <10% Acceptable: 10%-20% Poor: >20%
Classification	AUC^70^ Excellent: >0.90 Good: 0.80-0.89 Fair: 0.70-0.79 Poor: 0.60-0.69 Accuracy, sensitivity, specificity, PPV, NPV, and F1^71^ Excellent: >90% Good: 80%-90% Acceptable: 70%-79% Poor: <70% MAE and RMSE^77^ Excellent: <0.05 Good: 0.05-0.10 Fair: 0.10-0.20 Poor: >0.20 MSE^30,77^ Excellent : <0.0025 Good: 0.0025-0.01 Fair: 0.01-0.04 Poor: >0.04
Segmentation	DSC^72^ Excellent: >0.90 Good: 0.80-0.89 Fair: 0.70-0.79 Poor: <0.70 IoU/Jaccard index^73^ Excellent: >0.85 Good: 0.70-0.84 Fair: 0.50-0.69 Poor: <0.50 HD95^73^ Excellent: <2-3 mm or voxels Good: 3-5 mm or voxels Acceptable: 5-10 mm or voxels Poor: >10 mm or voxels Pixel-wise accuracy^74^ Excellent: >0.99 Good: 0.95-0.99 Fair: 0.90-0.94 Poor: <0.90 mAP^74^ Excellent: >0.80 Good: 0.60-0.79 Fair: 0.50-0.59 Poor: <0.50
NLP and foundation models metrics	BLEU^75^ Excellent (high overlap, near human-level translation): >0.40 Good (acceptable for clinical reporting or summarization): 0.30-0.39 Fair (domain-specific tasks): 0.20-0.29 Poor (low lexical match): <0.20 ROUGE Excellent (human-comparable for extractive tasks): >0.60 Good (typical for clinical summarization): 0.45-0.59 Fair (acceptable for abstractive tasks): 0.30-0.44 Poor: <0.30 METEOR^76^ Excellent (strong semantic match): >0.50 Good: 0.35-0.49 Fair: 0.25-0.34 Poor: <0.25 PPL^76^ Excellent (very fluent, human-like text): <20 Good (generally acceptable for medical NLP): 20-50 Fair (basic language modeling): 50-100 Poor (high uncertainty in word prediction): >100

These thresholds are intended as general guidelines. Acceptable thresholds may vary depending on clinical context, data set characteristics, and task complexity.

AUC = area under the curve; DSC = Dice similarity coefficient; HD95 = Hausdorff distance, 95th percentile; ICC = intraclass correlation coefficient; LoA = limits of agreement; MAE = mean absolute error; mAP = mean average precision; METEOR = metric for evaluation of translation with explicit ordering; MSE = mean squared error; NPV = negative predictive value; PPL = perplexity; PPV = positive predictive value; RMSE = root mean squared error; ROUGE = Recall-Oriented Understudy for Gisting Evaluation; other abbreviations as in Tables 1 and 2.

**TABLE 8:** Pitfalls and Considerations for Common Validation Metrics Across Classification, Segmentation, Language, and Multimodal Tasks

Metrics	Pitfalls and Considerations
Classification^71^	
Accuracy	May be misleading in imbalanced data sets (eg, detecting rare cardiac anomalies), as high accuracy can be achieved by always predicting the majority class.
Sensitivity and specificity	Should be balanced to avoid both undertreatment (low sensitivity) and prevent overdiagnosis (low specificity).
Precision	Is sensitive to data imbalance and does not account for false negatives.
F1 score	Harmonizes precision and recall, making it especially suitable for imbalanced data sets like those seen in rare cardiac conditions (eg, cardiac amyloidosis, arrhythmogenic right ventricular cardiomyopathy).
AUC-ROC	Evaluates a model’s ability to distinguish between classes across all threshold values; this may be misleading in highly imbalanced data sets, as it gives equal weight to both classes and is less informative when the focus is on identifying true positives accurately in minority classes.
AUC-PR	Is preferred when positive cases are rare, as it emphasizes performance on the positive class.
MAE, MSE, RMSE	These metrics quantify prediction error in continuous variables, such as estimating left ventricular ejection fraction. MAE is less sensitive to outliers, whereas MSE and RMSE penalize larger errors more heavily, making them appropriate when high-risk mispredictions could lead to patient harm.
Segmentation^80^	
DSC and IoU	High DSC indicates accurate anatomical boundary recognition, which is essential for downstream volume and function analysis. DSC and IoU can be less informative when dealing with large background regions, as small segmentation errors in minority classes can be overshadowed and when predicting segments that are very small.
Hausdorff distance	Quantifies the maximum spatial discrepancy between the boundaries of 2 shapes or segmentations, making it a valuable metric for evaluating how closely an automated cardiac image segmentation (eg, of the myocardium or chambers) aligns with expert manual annotations.
Pixel-wise accuracy	Can be misleading in imbalanced cases where background dominates (eg, segmenting the myocardium within a large thoracic field); high accuracy can mask poor segmentation of clinically relevant regions. Also, it is not informative about the quality of boundaries or regional coherence.
mAP	mAP measures detection accuracy across classes and is widely applied in multilabel segmentation, eg, distinguishing between atrial, ventricular, and pericardial compartments in MRI. mAP requires careful definition of true positives and matching criteria, which may be ambiguous in artifact-prone regions of cardiac anatomy.
NLP and foundation models metrics^81,82^	
BLEU, ROUGE, METEOR scores	These metrics assess the fidelity of clinical text, such as echocardiography report structure or automatic impression generation. BLEU and ROUGE compare n-gram overlaps, whereas METEOR accounts for synonyms and paraphrasing. BLEU is insensitive to semantic correctness and paraphrasing and can penalize valid medical rephrasing (eg, “left ventricular systolic dysfunction” vs “reduced ejection fraction”). ROUGE does not assess factual correctness and may reward verbosity over clinical precision. METEOR does not fully account for medical domain-specific synonyms unless customized and can be computationally more intensive.
PPL	Measures how well a language model predicts the next word in a sentence. Lower PPL indicates better fluency and contextual understanding. PPL is not aligned with clinical accuracy or relevance; a model can have low perplexity but still generate factually incorrect or unsafe content; also, it does not assess alignment with reference texts, which is important in structured reporting contexts.

PR = precision recall; ROC = receiver-operating characteristic; other abbreviations as in Tables 1 and 7.

**TABLE 9:** List of Technical Metrics, Clinical Utility Metrics and Health Care Economics Metrics for Evaluation of AI Solutions in Health Care

Clinical Utility Metrics (Patient Benefits)	Economics Metrics (Health Care Benefits)
Impact of quantification error on disease grading Consequences of false positive and false negative diagnosis Attention to potential bias on minority and subgroups Clinical decision support value using DCA or NCB	Cost-effectiveness analysis of new clinical workflow with the proposed technique Short and long-term implementation costs Overall return on investment

DCA = decision curve analysis; NCB = net clinical benefit.

**TABLE 10:** Common Study and Model Limitations in Cardiovascular Imaging AI

	Specific Limitations	Examples	Assessment Methods
Data limitations	Scope and representativeness; data quality; label quality	Small sample sizes, single-center design, limited demographic diversity; missing data, noise, artifacts, resolution variations, protocol heterogeneity; interobserver variability, ground truth unavailability	Sample size calculations, demographic analysis, data completeness metrics, data quality assessments, annotation agreement statistics
Methodological limitations	Model assumptions; algorithmic constraints; methodology simplifications	Linearity/independence assumptions, data distribution assumptions; architecture capacity limits, optimizer constraints; image down sampling (spatial/temporal), preprocessing shortcuts	Assumption testing, model diagnostic plots, architecture ablation studies, preprocessing sensitivity analysis
Generalizability boundaries	Population limits; technical limits; clinical context limits	Different demographics, disease patterns, geographic regions; scanner manufacturers, protocols, reconstruction algorithms; health care settings, user expertise, workflow integration	External validation across populations, multisite testing, protocol variation analysis, workflow integration studies
Advanced model limitations	Interpretability and explainability challenges; hallucination risks; computational constraints	"Black box" decision-making, feature attribution difficulty; LLM factual errors, GenAI controllability issues; high inference costs, latency requirements	Explainability analysis, uncertainty quantification, hallucination detection, computational profiling

GenAI = generative artificial intelligence; other abbreviations as in Table 1.

**TABLE 11:** Common Types of Bias in Cardiovascular Imaging AI

	Description	Examples	Assessment Methods	Mitigation Strategies
Selection bias	Systematic differences in imaged populations	Referral pattern variations by demographics, insurance-based access differences	Demographic analysis, referral pattern assessment, population comparison	Multisite recruitment, diverse referral sources, targeted outreach
Representation bias	Inadequate demographic diversity in training	Underrepresentation of minorities, age groups, or geographic regions	Subgroup sample size analysis, demographic distribution comparison	Data augmentation, targeted recruitment, synthetic data generation
Measurement bias	Systematic imaging quality/protocol differences across groups	Scanner quality variations by hospital type, contrast protocol differences	Protocol standardization analysis, quality metrics by subgroup, technical parameter assessment	Standardized protocols, quality controls, technical harmonization
Annotation bias	Expert interpretation varies by patient characteristics	Reader bias based on patient demographics or clinical presentation	Inter-reader variability analysis by demographics, bias detection studies	Multiple reader consensus, bias awareness training, blinded annotations
Algorithmic bias	Model architecture/training favors certain populations	Feature selection encoding demographic information, optimization bias	Fairness metrics (demographic parity, equalized odds, calibration), subgroup performance analysis	Fairness-aware training, adversarial debiasing, balanced optimization
Historical bias	Training data reflects past health care disparities	Legacy data with embedded disparities, outdated clinical practices, temporal changes in care standards	Temporal analysis, outcome disparity assessment, historical trend evaluation	Contemporary data prioritization, bias-aware preprocessing, disparity correction, inclusive data governance

Abbreviation as in Table 1.

## Panel 1. DL Architectures in Cardiovascular Imaging

- CNNs: excel at learning hierarchical features from imaging data
- Specialized architectures such as U-net and nnU-net: tailored toward cardiac segmentation in multiple contexts, including echocardiography, CT, and MRI
- RNNs: suited for analyzing time-series data like ECG signals
- GANs and diffusion models: create synthetic cardiac images and enabling data augmentation for rare disease modeling
- Graph neural networks: relational modeling capabilities that capture the structure of graph-structured data (eg, 3-dimensional meshes or coronary trees
- Autoencoders: valuable for anomaly detection in cardiovascular imaging
- Vision Transformers (ViTs): powerful alternatives as they may go beyond neighboring patches of the input data (eg, groups of pixels in the region of interest) and take advantage of more distant data patches to perform prediction, therefore improving image and text analysis tasks

(Al-Hammuri, Gebali, Kanan, & Chelvan, 2023; Ansari, Mourad, Qaraqe, & Serpedin, 2023; Ferreira, Lau, Salaymang, & Arnaout, 2025; Hampe et al, 2024; Hinck et al, 2025; Liang et al, 2024; Lomoio et al, 2025; Mhamdi, Dammak, Cottin, & Dhaou, 2022; Rawshani et al, 2025; Sakli et al, 2022; Schilling, Unterberg-Buchwald, Lotz, & Uecker, 2024; Skandarani, Lalande, Afilalo, & Jodoin, 2022; Staffini, Svensson, Chung, & Svensson, 2023; Vaid et al, 2023; Xu & Wu, 2024; Yamashita, Nishio, Do, & Togashi, 2018; Yoon et al, 2023) relate to the content in the panel and are identified in the Supplemental References.

## Panel 2. Applications of Language Models to Cardiovascular Imaging

- LLMs, whether foundational or domain-adapted, can encode a broad spectrum of echocardiographic phenotypes directly from free-text reports. For example, contrastive learning enabled the alignment of a text encoder adapted for echocardiography reports with a vision encoder built for corresponding echocardiograms. This approach enabled the generation of latent representations that captured diagnostic reasoning without explicit supervision.
- LLMs may also be used to structure large volumes of free-text imaging reports, enabling scalable tabular curation for downstream supervised learning tasks. For example, HeartDX-LM, open-source LLMs, such as Llama2-13b and 70b, were fine-tuned to automate the extraction of unstructured echocardiographic reports into tabular datasets. These efforts facilitate the creation of large institutional datasets and support harmonization across multicenter studies, an essential step for developing robust and generalizable machine learning models.
- End-to-end automation of cardiovascular imaging reports through text decoders includes, for example, applications to chest radiographs and 3-dimensional CT imaging. These models may also provide interactive functionality, allowing clinicians to query or highlight imaging using natural language in real-time.
- On a technical note, pre-trained LVLMs or other vision encoder architectures represent a powerful way to initialize models when dealing with limited datasets, by enabling efficient fine-tuning and supporting their downstream generalizability.

(Arnaout, 2024; Blankemeier et al, 2024; Christensen, Vukadinovic, Yuan, & Ouyang, 2024; Lu et al, 2024; Shankar et al, 2024; Tanno et al, 2025; Vukadinovic et al, 2024) relate to the content in the panel and are identified in the Supplemental References.

## Panel 3. Supervised and Unsupervised Learning

- For *supervised* DL models, a framework that is robust and contains a degree of explainability is important; notable examples include gradient boosting machines (eg, XGBoost coupled with SHAPley values for prediction of myocardial infarction) attentive interpretable tabular learning (eg, TabNet, which has been used to in prediction of cardiac arrest prediction), and feedforward neural networks (eg, multilayer perceptron for ambulatory laboratory results).
- For *unsupervised* DL models, pattern recognition and detection of outliers is important, making autoencoders a valuable resource. Furthermore, explicit feature engineering (eg, standardization of values) can help mitigate the effects of high interobserver variability and modality-specific artifacts, improving model reliability and aiding clinical translation in heterogeneous cardiovascular datasets.

(Arik & Pfister, 2019; Behnam et al, 2025; Moore & Bell, 2022; Nguyen & Byeon, 2023; Titar & Ramanathan, 2024) relate to the content in the panel and are identified in the Supplemental References.

## Panel 4. DL Architectures in Cardiac Image Segmentation and Data Augmentation

- CNN-based DL architectures, importantly U-net and nnU-net and their variants, state-of-the-art cardiac segmentation due to their ability to capture fine-grained anatomical details while preserving contextual information; also serving as top-performing methods in multiple large-scale data challenges.
- For modalities involving real-time or multi-phase data (eg, cine MRI or Doppler echocardiography), models such as 3-dimensional U-net or recurrent variants (eg, multiscale attention-guided U-net architectures) are better suited to handle temporal dynamics.
- Integration of attention mechanisms and residual connections can further enhance model resilience to image noise and anatomical variability. Additionally, segmentation pipelines must be carefully validated against clinically meaningful reference standards to account for high inter-observer variability.
- ViTs, such as the Swin Transformer, have emerged as powerful alternatives to traditional CNNs by leveraging self-attention mechanisms to model long-range dependencies and global contextual relationships, an advantage particularly beneficial in segmenting complex cardiac structures with variable shape, size, and location.
- Attention-enhanced CNNs, which incorporate attention gates or modules into U-Net-like architectures, further improve segmentation accuracy by selectively emphasizing salient regions and suppressing background noise, making them well-suited for adapting to artifact-prone and heterogeneous images.
- GANs, variational encoders, and diffusion models have been increasingly used in cardiac image segmentation to augment training datasets, synthesize realistic anatomical variations, and enhance segmentation accuracy in low-data or imbalanced scenarios. For example, GANs can produce realistic echocardiographic images of rare congenital cardiac defects and enhance low-resolution cardiac magnetic resonance images for better segmentation fidelity.
- Diffusion models offer better control over image generation and often yield more anatomically accurate and realistic results in cardiovascular imaging tasks, unlike GANs, which can suffer from mode collapse (producing limited variation) and training instability.

(Alnasser et al, 2024; Bernard et al, 2018; Cui, Yuwen, Jiang, Xia, & Zhang, 2021; Fakhfakh, Sarry, & Clarysse, 2025; Islam, Qaraqe, & Serpedin, 2024; Leclerc et al, 2019; Streiffer, Levin, Witschey, & Anyanwu, 2024; Suha et al, 2025; Vafaeezadeh, Behnam, & Gifani, 2024; Zhao, Wei, & Wong, 2022; H. Zhou et al, 2024) relate to the content in the panel and are identified in the Supplemental References.

## Panel 5. Foundation Models and Multimodal Models in Cardiovascular Imaging

- A key decision in selecting an LLM for cardiovascular imaging is whether to use a general-purpose model or one fine-tuned on biomedical corpora.
  - Fine-tuned models offer advantages like domain-specific vocabulary and improved accuracy in tasks such as information extraction, summarization, and question answering.
  - These models are typically trained on clinical texts (eg, radiology reports, peer-reviewed published reports), which helps reduce factual errors.
  - However, fine-tuned models may be smaller, less flexible, less robust to informal inputs, and often require further tuning for specific institutional needs.
  - It’s also important to consider the model’s ability to process multimodal inputs (eg, text and imaging metadata) and adapt to local reporting templates.
  - In cardiovascular imaging, recent efforts have developed domain-specific LLMs for generating automated echocardiography draft reports in low-data settings.
- GPT-4V has shown success in chest radiograph interpretation, particularly when using few-shot learning.
- Multimodal models like MAIRA-2 and EchoCLIP improve explainability and accuracy by combining text and image data, using techniques like grounding and bounding boxes to highlight findings, and can even identify patients with prior surgeries or implanted devices.

(Bannur et al, 2024; Chao et al, 2025; Christensen et al, 2024; Nazi & Peng, 2024; Neveditsin, Lingras, & Mago, 2025; Subramaniam et al, 2025; Y. Zhou et al, 2024) relate to the content in the panel and are identified in the Supplemental References.

## Panel 6. Limitations for Validation Metrics in Multimodal Models

- Combined evaluation metrics assess both visual interpretation and text generation quality. In multimodal models, diagnostic correctness from imaging (eg, DSC, IoU, and mAP) is evaluated alongside textual fidelity (eg, BLEU, AUC, and ROUGE jointly).
- Additionally, there are some specific evaluation metrics for multimodal models, including cross-modal retrieval metrics such as mean reciprocal rank used in EchoCLIP to assess retrieval of matching views or reports.
- Other examples include CLIPScore, which measures the cosine similarity between image and text embeddings in a joint space (used for image captioning or report generation), and image-text matching, which is a binary metric indicating whether a model can correctly identify if an image-text pair belongs together.

(Christensen et al, 2024; Hessel, Holtzman, Forbes, Bras, & Choi, 2022; Jin, Hou, Jin, Yuan, & Du, 2024) relate to the content in the panel and are identified in the Supplemental References.

## Panel 7. Evaluating Data Quality by Task

- Segmentation Tasks
  - AI models must generalize across scanners from different manufacturers.
  - Differences in protocols (eg, slice thickness, contrast dose) can affect model performance.
  - Models trained on one scanner may underperform when applied to another without adaptation.
  - Techniques like domain adaptation, data harmonization (eg, ComBat), and multi-institutional training help address this.
  - Cardiovascular imaging variability arises from patient movement, hardware differences, and acquisition parameters.
  - Models must be robust to low signal-to-noise ratios, variable resolution, and motion artifacts.
  - Solutions include image denoising, super-resolution, and training with quality-degraded inputs to improve resilience.
- NLP and Foundation Models Tasks
  - Language models should be validated across specialties (eg, cardiology) and care settings (eg, inpatient vs outpatient).
  - Domain shifts (eg, note style, terminology, or sparsity) can degrade performance.
  - Clinical language is highly variable due to abbreviations, shorthand, and provider habits.
  - Robust LLMs must adapt to different writing styles, detail levels, and specialty-specific terms.
  - Fine-tuning on targeted domains and generating synthetic reporting can enhance model reliability.
- Multiple Data Sources/Multimodal AI Tasks
  - Multimodal models should leverage redundancy (eg, strong clinical history can offset weak image quality).
  - Training with noisy, real-world data improves generalization and resilience.
  - Models should be tested on incomplete input scenarios (eg, missing or low-quality modalities).

For example, a cardiac amyloidosis prediction model using echocardiography and EMR text should remain reliable if echocardiography input is degraded.
  - External validation is essential using datasets with similar modality combinations.
  - Validation datasets should vary in geography, patient demographics, and clinical settings. This ensures that multimodal fusion logic is not overfit to specific training data correlations.

(Christensen et al, 2024; Fathi et al, 2020; Priya et al, 2023; Simon, Ozyoruk, Gelikman, Harmon, & Turkbey, 2024; Zhao et al, 2022) relate to the content in the panel and are identified in the Supplemental References.

## Panel 8. AI Model Interpretability, Explainability and Bias/Fairness by Task

- Segmentation Tasks – Sources of bias may also come from additional image-related factors, such as image quality or resolution specific to the acquisition site, device, and/or operator. Bias mitigation typically involves image pre-processing and post-processing, to compensate for both underrepresentation of specific patient groups and differences in image characteristics. Notable techniques include oversampling the minority classes, data augmentation with arbitrary image transformations, decomposition of images into smaller patches, and generation of realistic synthetic images (state-of-the-art methods for this consisting of Generative Adversarial Networks and Diffusion Models).
- NLP and Foundation Models Tasks – Current LLMs trained on clinical notes or biomedical literature may internalize stereotypes or disparities, suffer from unverified training data, and can produce plausible but not accurate information, with an increased risk that these limitations accelerate due to exponentially growing use. Recently proposed human evaluation frameworks are a first necessary step towards trusting these models for clinical applications, although these models require advanced governance efforts.
- Multimodal – These types of data may be partially redundant depending on the patient or disease, but may weight differently in taking the decision (for example, a scalar clinical variable only represents one value to be potentially combined with the information from all pixels of an image, or the data from each modality may not be of the same quality), although recent techniques such as transformers are able to optimize the distribution of information into pieces during their tokenization process.

(Chawla, Bowyer, Hall, & Kegelmeyer, 2002; Comeau, Bitterman, & Celi, 2025; Kazerouni et al, 2023; Singhal et al, 2023; Thirunavukarasu et al, 2023; Vaswani et al, 2017; Yi, Walia, & Babyn, 2019) relate to the content in the panel and are identified in the Supplemental References.

## Panel 9. Quantifying Uncertainty

- Popular methods in health care applications include Bayesian inference and its extensions such as Dempster-Shafer’s theory, Monte Carlo simulation, Fuzzy systems, or broader concepts such as imprecise probability.
- Recent recommendations distinguish the uncertainty estimation methods according to the type of AI model used, such as single deterministic methods (relying on a single forward pass), Bayesian methods (formulating the relation between inputs and outputs in probabilistic terms), ensemble methods (which consist of the fusion of multiple deterministic networks), and test-time augmentation methods (which generate multiple predictions by augmenting the input data).
- An alternative version distinguishes between methods equipping the decision predicted by the AI model with an uncertainty range, and those defining beforehand an expected level of certainty and delivering a decision that fits this range.

(Gawlikowski et al, 2023; Hüllermeier & Waegeman, 2021; Seoni et al, 2023) relate to the content in the panel and are identified in the Supplemental References.

## Panel 10. Cost-Effectiveness and Economic Evaluation

- A common method is cost-effectiveness analysis (CEA), which calculates the incremental cost-effectiveness ratio (ICER)—the additional cost per unit of health benefit gained, often expressed as cost per quality-adjusted life year (QALY). AI may contribute to the cost-effectiveness by detecting and stratifying disease earlier and more accurately, recommending personalized treatment based on big data, and optimizing the hospital resource distribution and scheduling.
- Budget impact analysis (BIA) can estimate the short- and long-term financial implications of implementing AI tools within specific healthcare settings. In the context of AI solutions and AI-powered medical devices, short-term financial costs include device hardware, AI computing equipment, licensing, personnel recruitment and training, and regulatory compliance. Long-term costs should consider energy use, cabin and water footprint, especially for computationally intensive AI applications like imaging.
- Return on investment (ROI) provides a complementary measurement to calculate the economic gain achieved from investing in an AI solution, typically expressed as a percentage of net profit over total investment. Benefits may include the savings of personnel by reduced workload via AI automation, such as for image analysis, reporting, and medical procedures; shorter and more efficient examinations; and higher diagnostic accuracy, translating into overall operational savings and lower healthcare burdens at the population level.

(Hanneman et al, 2025) relate to the content in the panel and are identified in the Supplemental References.

## HIGHLIGHTS

- Rapid growth in AI for cardiovascular imaging highlights the need for structured reporting guidance.
- PRIME 2.0 introduces a cardiac imaging-specific, expert-derived checklist across 7 key study domains across the lifecycle of AI research.
- The framework fosters transparency, reproducibility, and alignment with evolving AI technologies and clinical needs.
- Comprising essential and advanced elements, the 2-tiered checklist is designed specifically to support flexible and scalable integration into cardiac imaging research across diverse health care settings.

## APPENDIX

For a supplemental tables and references, an Essential Checklist Template, and an Extended Checklist Template, please see the online version of this paper.

## ABBREVIATIONS AND ACRONYMS

AI: artificial intelligence
CNN: convolutional neural network
DCA: decision curve analysis
DL: deep learning
ECG: electrocardiogram
GAN: generative adversarial network
LLM: large language model
LVLM: large vision-language model
ML: machine learning
MRI: magnetic resonance imaging
NCB: net clinical benefit
NLP: natural language processing
RCT: randomized controlled trial
RNN: recurrent neural network
