# Supplementary material for: PRIME 2.0: Proposed Requirements for Cardiovascular Imaging-Related Multimodal-AI Evaluation: An Updated Checklist
Source: JACC Cardiovasc Imaging. Author manuscript; Available in PMC 2026 Oct 1. (PMC13628111; doi:10.1016/j.jcmg.2025.08.004)
Supplement: Supplementary file 2 [file NIHMS2210662-supplement-Supplementary_Table_7.docx]

## Essential Reporting Checklist for Researchers

| **Section** | **Checklist item** | **Page #** |
| --- | --- | --- |
| **1** | **Designing an AI Study in Cardiovascular Imaging** |  |
| *1.1* | *Appropriateness of applying AI* |  |
| a | Describe the need for applying AI |  |
| b | Determine the appropriateness of applying AI |  |
| *1.2* | *Study objectives, input data type, and prediction target* |  |
| a | Explain the AI task and the likely deployment context |  |
| b | Describe the input data, number of training/test examples |  |
| c | Specify model supervision type |  |
| d | Describe the nature of the model’s output and what it represents |  |
| *1.3* | *Design of the AI study* |  |
| a | Describe the study design |  |
| b | Describe data origin |  |
| c | Describe if impact analysis was included |  |
| **2** | **Data Format and Preprocessing** |  |
| *2.1* | *Clinical characteristics of the study cohort* |  |
| a | Present the age, sex, and race/ethnicity distributions of the cohort(s) |  |
| b | Summarize key clinical, treatment, and imaging characteristics of the cohort(s) |  |
| c | Compare summary statistics of cases and controls |  |
| **3** | **Selection of AI Methods and Applications** |  |
| *3.1* | *Selecting appropriate AI methods and applications: Clearly define data composition (structured/unstructured)* |  |
| *3.2, 3.3, 3.4* | *Training strategies: Describe the AI method/application used, with rationale for clinical task fit* |  |
| **4** | **Model Assessment** |  |
| *4.1* | *Importance of model assessment: Describe the evaluation approach and how it addresses the clinical question* |  |
| *4.2* | *Technical performance metrics* |  |
| a | Report relevant performance metrics (F1-score, IoU, BLEU, etc.) |  |
| b | Justify metric selection based on task characteristics |  |
| c | Describe manual annotation process, reference-standard prep, and observer variability |  |
| **5** | **Clinical Evaluation** |  |
| *5.1* | *Importance of clinical evaluation: Describe potential clinical impact of misclassification* |  |
| *5.2* | *Clinical validation: Provide evidence of clinical validation* |  |
| **6** | **Best Practices for Replicability** |  |
| *6.1* | *Importance of transparency and open science principles* |  |
| a | Ensure data sharing follows FAIR principles |  |
| b | Report on training data representativeness |  |
| **7** | **Reporting of Limitations, Biases, and Alternatives** |  |
| *7.1* | *Acknowledging study and model limitations* |  |
| a | Discuss key limitations (data, methodology, generalizability) |  |
| b | Report sensitivity analyses and model-specific issues |  |
| *7.2* | *Discussing study strengths: Articulate strengths (methodological rigor, dataset quality, innovation, clinical relevance)* |  |
