# Supplementary material for: PRIME 2.0: Proposed Requirements for Cardiovascular Imaging-Related Multimodal-AI Evaluation: An Updated Checklist
Source: JACC Cardiovasc Imaging. Author manuscript; Available in PMC 2026 Oct 1. (PMC13628111; doi:10.1016/j.jcmg.2025.08.004)
Supplement: Supplementary file 3 [file NIHMS2210662-supplement-Supplementary_tables_and_references.docx]

## Supplemental Table 1. Major Guidelines for AI in Medical Research

| **Guideline (Abbreviation)** | **Year** | **Primary Focus** | **Core Objective** | **Intended Users** | **Key AI-Specific Considerations** | **Reference** |
| --- | --- | --- | --- | --- | --- | --- |
| **SPIRIT‑AI** | 2020 Sep | RCT protocols | Protocol transparency | Researchers, Ethics committees | AI intervention definition; workflow; I/O data; error‑analysis plan; code availability | Rivera et al., Nat Med, 2020 (1) |
| **CONSORT‑AI** | 2020 Sep | RCT final reports | Trial transparency | Researchers, Journal editors | AI version; data eligibility; error‑analysis; code availability | Liu et al., Nat Med, 2020 (2) |
| **DECIDE‑AI** | 2022 May | Early clinical evaluation | Usability & safety assessment | Researchers, Clinicians | Human factors; usability; workflow; safety | Vasey et al., Nat Med, 2022 (3) |
| **STARD‑AI** | 2020 Jun | Diagnostic accuracy studies | Reporting transparency | Researchers, Developers | Development/validation; datasets; performance metrics | Sounderajah et al., Nat Med, 2020 (4) |
| **TRIPOD+AI** | 2024 Apr | Prediction/model development studies | Transparency & reproducibility | Researchers, Developers | Fairness eval; code/data sharing; regression & ML | Collins et al., BMJ, 2024 (5) |
| **TRIPOD‑LLM** | 2025 Jan | LLM-based studies | Reporting transparency | Researchers, Developers | Prompt design; output & human evaluation | Gallifant et al., Nat Med, 2025 (6) |
| **CLAIM** | 2020 Mar; 2024 Jul | Medical imaging AI studies | Transparency & reproducibility | Researchers, Developers | Image acquisition; preprocessing; reference standards; terminology | Mongan et al., Radiol Artif Intell, 2020/2024 (7) |
| **PROBAST+AI** | 2025 Mar | Bias in prediction models | Bias & applicability assessment | Researchers, Reviewers, Developers | Domain signalling items; fairness | Moons et al., BMJ, 2025 (8) |
| **CHEERS‑AI** | 2024 May | Health economic evaluations | Cost‑effectiveness analysis | Researchers, Health economists | Economic transparency; 10 AI-specific items | Elvidge et al., Value Health, 2024 (9) |
| **MI‑CLAIM** | 2020 Sep | Clinical AI modelling | Clinical impact & reproducibility | Researchers, Developers | Design; optimization; metrics; transparency tiers | Norgeot et al., Nat Med, 2020 (10) |
| **MINIMAR** | 2020 Dec | Medical AI reporting | Standardization & comparability | Researchers, Developers | Population; demographics; architecture; evaluation | Hernandez-Boussard et al., JAMIA, 2020 (11) |
| **PRISMA‑AI** | 2023 Jan | Systematic reviews/meta‑analyses | Reporting transparency | Researchers, Reviewers | Search strategy; AI-specific bias; study quality | Cacciamani et al., Nat Med, 2023 (12) |
| **Radiomics Quality Score** | 2017 | Radiomics studies | Methodological quality evaluation | Researchers, Radiologists | Rigor; cost-effectiveness; clinical utility | Lambin et al., Nat Rev Clinic Onco 2017 (13) |
| **FUTURE‑AI** | 2025 | AI lifecycle integration | Trustworthiness | Developers, Regulators, Clinicians | Fairness; universality; traceability; usability; robustness; explainability | Lekadir et al., BMJ, 2025 (14) |
| **WHO Guidance** | 2024 | AI ethics & governance | Ethical/human‑rights compliance | Governments, Businesses, Providers | Autonomy; fairness; accountability; LLM risks | WHO, 2024 (15) |
| **OECD Principles** | 2019 | National AI policy | Responsible innovation | Policymakers | Inclusive growth; rights; values; international cooperation | OECD, 2019 (16) |

## Supplemental Table 2 Taxonomy of AI Models and Evaluation Strategies in Cardiac Imaging

|  | Example 1:  rECHOmmend (17) | Example 2:  EchoNet-Dynamic (18) | Example 3:  SwedeHF Phenotyping (19) |
| --- | --- | --- | --- |
| Clinical task  *What clinical problem is the model intended to address?* | Opportunistic detection of undiagnosed SHD from ECG | Automated estimation of LVEF from single-view echocardiographic videos | Identification of HF phenotypes for improved risk stratification and treatment targeting |
| Deployment context  *Who should use it, and when and how should it be used in the clinical workflow?* | Used by clinicians to identify patients with high-risk ECGs who may benefit from echocardiography | Used by cardiologists and sonographers during echocardiogram interpretation | Used by researchers and potentially clinicians to better assess risk and treatment in patients with heart failure |
| Model development strategy  *New or fine-tuned model or external validation of a previously published model?* | De novo model development | De novo model development | De novo model development |
| Input modality  *What kind of data does the model take as input?* | 12-lead ECG signals | Apical 4-chamber view grayscale echocardiographic videos | Tabular EHR data from the Swedish Heart Failure Registry |
| Supervision type  *Supervised, unsupervised, or hybrid approach?* | Supervised | Supervised | Hybrid: supervised (survival prediction) + unsupervised (identifying HF phenotypes) |
| Output type  *What output does the model produce?* | Binary labels for individual SHD conditions and a composite SHD | Continuous monoplane LVEF and frame-by-frame segmentation masks | Continuous 1-year survival probabilities + HF phenotype cluster membership |
| AI = artificial intelligence; ECG = electrocardiogram; EHR = electronic health record; HF = heart failure; LVEF = left ventricular ejection fraction; SHD = structural heart disease | | | |

## Supplemental Table 3 Examples of challenges and mitigation strategies that can be applied during data preprocessing

| **Challenge** | **Possible mitigation strategies during preprocessing** |
| --- | --- |
| Variability across different views | Include only relevant views |
| Frame rate variability | Adjust the frame rate to make it uniform across all videos |
| Variability in the scaling of images | Adjust the image size to achieve uniform scaling across all images |
| Irrelevant information in the image | Crop the image to exclude irrelevant pixels |
| Inter-vendor variability | Perform subgroup analysis to ensure that the model is not affected by the difference between the machines of different vendors |
| Model generalizability | Train and validate the model using data from multiple institutions |

## Supplemental Table 4: Representative examples of language model-enabled applications across medical imaging.

3D: 3-dimensional; AI: artificial intelligence; CNN: convolutional neural network; CT: computed tomography; EHR: electronic health record; LLM = large language model; (L)VLM = (large) vision-language model; MRI: magnetic resonance imaging.

| **Summary** | **Description** | **Example** | **Language Model / Encoder** | **Translation to Broader CV Imaging** |
| --- | --- | --- | --- | --- |
| **EchoCLIP:**  A contrastive learning model aligning single-view echocardiography videos and corresponding reports (20) | Trained on over 1 million echocardiographic video-report pairs to learn latent representations capturing diagnostic reasoning without explicit supervision | May enable zero-shot identification of diverse phenotypes and supports scalable, label-efficient phenotyping in echocardiography. | Small domain-adapted encoders (custom transformer) | The same framework can be extended to multi-view cardiovascular imaging modalities, including CT angiography or cardiac MRI for automated reporting and phenotype extraction. |
| **EchoPrime:**  Multi-view vision-language model for comprehensive echocardiography interpretation (21) | Multi-view, video-based vision-language foundation model trained on over 12 million echocardiogram video-report pairs that enables holistic, anatomically-informed interpretation of comprehensive echocardiography studies through contrastive learning and retrieval-augmented integration. | May facilitate holistic interpretation of echocardiographic studies, potentially improving diagnostic accuracy and efficiency. |  |  |
| **HeartDx-LM:**  LLM fine-tuned for structuring echocardiographic reports (22) | Employs Llama2-13b and 70b models to convert unstructured echocardiography reports into structured datasets across multiple institutions | Streamlines data curation for supervised learning and supports harmonization across multi-center studies | Large LLM (Llama2-13b, Llama2-70b) | Supports scalable data extraction across institutions. Can be extended to additional imaging modalities, enabling harmonized AI pipelines for CT, MRI, and nuclear imaging. |
| **Flamingo-CXR:**  Vision-language model for automated chest X-ray report generation (23) | Specialized VLM fine-tuned on radiology data to generate clinically relevant chest radiograph reports | Demonstrates potential for automating report generation in radiology, with implications for similar applications in cardiovascular imaging | Large VLM (DeepMind Flamingo), includes decoder | Can be adapted to generate structured cardiovascular imaging reports from CT or MRI by integrating domain-specific visual encoders. |
| **Merlin:**  3D vision-language foundation model for CT reporting (24) | A 3D vision-language foundation model trained on over 15,000 abdominal CT scans, integrating 6 million images, 1.8 million EHR diagnosis codes, and 6 million radiology report tokens | Enables automated interpretation of 3D CT scans, including zero-shot classification, phenotype prediction, and report generation, by leveraging both structured EHR data and unstructured radiology reports. | 3D CNN-based vision encoder, and transformer-based(text) decoder | Adaptable to various 3D medical imaging modalities beyond abdominal CT, facilitating scalable and generalizable AI applications in clinical settings. |
| EchoCLIP = Echocardiography Contrastive Language-Image Pretraining, EchoPrime = Multi-view foundation model for comprehensive echocardiography interpretation, HeartDx-LM = Large Language Model fine-tuned for echocardiographic report structuring, Flamingo-CXR = Vision-Language Model specialized for chest X-ray report generation, Merlin = 3D Vision-Language Foundation Model for CT interpretation, LLM = Large Language Model, VLM = Vision-Language Model, CT = Computed Tomography, MRI = Magnetic Resonance Imaging, EHR = Electronic Health Record, CNN = Convolutional Neural Network | | | | |

## Supplemental Table 5 The FAIR Guiding Principles

**Findable**

F1. (meta) data are assigned a globally unique and persistent identifier

F2. data are described with rich metadata (defined by R1 below)

F3. metadata clearly and explicitly include the identifier of the data it describes

F4. (meta) data are registered or indexed in a searchable resource

**Accessible**

A1. (meta) data are retrievable by their identifier using a standardized communications protocol

A1.1 the protocol is open, free, and universally implementable

A1.2 the protocol allows for an authentication and authorization procedure, where necessary

A2. metadata are accessible, even when the data are no longer available

**Interoperable**

I1. (meta) data use a formal, accessible, shared, and broadly applicable language for knowledge representation.

I2. (meta) data use vocabularies that follow FAIR principles

I3. (meta) data include qualified references to other (meta) data

**Reusable**

R1. meta (data) are richly described with a plurality of accurate and relevant attributes

R1.1. (meta) data are released with a clear and accessible data usage license

R1.2. (meta) data are associated with detailed provenance

R1.3. (meta) data meet domain-relevant community standards

## Supplemental Table 6. Major Data Repositories

| Platform | Code Sharing | Data Sharing | DOI Support | Key Features | Link |
| --- | --- | --- | --- | --- | --- |
| GitHub | ✔ Yes | ✕ Limited | ✕ (via Zenodo) | Popular for code collaboration and version control. Integrates with Zenodo. | [github.com](https://github.com/) |
| Zenodo | ✔ Yes | ✔ Yes | ✔ Yes | Archives code and data; issues DOIs; integrates directly with GitHub. | [zenodo.org](https://zenodo.org/) |
| Figshare | ✔ Yes | ✔ Yes | ✔ Yes | Used for sharing datasets, figures, and software as supplementary material. | [figshare.com](https://figshare.com/) |
| Dryad | ✕ (Code discouraged) | ✔ Yes | ✔ Yes | Focused on curated scientific datasets, especially in life sciences. | [datadryad.org](https://datadryad.org/) |
| OSF (Open Science Framework) | ✔ Yes | ✔ Yes | ✔ Yes | Project-based platform for sharing all research components. | [osf.io](https://osf.io/) |
| GitLab | ✔ Yes | ✕ Limited | ✕ | Similar to GitHub; often used for private or institutional repositories. | [gitlab.com](https://gitlab.com/) |
| Dataverse | ✕ (Primarily for data) | ✔ Yes | ✔ Yes | Hosted by institutions; structured metadata and versioning for datasets. | [dataverse.org](https://dataverse.org/) |
| Mendeley Data | ✕ (Primarily for data) | ✔ Yes | ✔ Yes | Structured data sharing platform; integrates with Elsevier journals. | data.mendeley.com |

**Supplementary Reference**

1.	Cruz Rivera S, Liu X, Chan A-W et al. Guidelines for clinical trial protocols for interventions involving artificial intelligence: the SPIRIT-AI extension. Nature Medicine 2020;26:1351-1363.

2.	Liu X, Cruz Rivera S, Moher D et al. Reporting guidelines for clinical trial reports for interventions involving artificial intelligence: the CONSORT-AI extension. Nature Medicine 2020;26:1364-1374.

3.	Vasey B, Clifton DA, Collins GS et al. DECIDE-AI: new reporting guidelines to bridge the development-to-implementation gap in clinical artificial intelligence. Nature Medicine 2021;27:186-187.

4.	Sounderajah V, Ashrafian H, Aggarwal R et al. Developing specific reporting guidelines for diagnostic accuracy studies assessing AI interventions: The STARD-AI Steering Group. Nat Med 2020;26:807-808.

5.	Collins GS, Moons KGM, Dhiman P et al. TRIPOD+AI statement: updated guidance for reporting clinical prediction models that use regression or machine learning methods. Bmj 2024;385:e078378.

6.	Gallifant J, Afshar M, Ameen S et al. The TRIPOD-LLM reporting guideline for studies using large language models. Nature Medicine 2025;31:60-69.

7.	Mongan J, Moy L, Kahn CE. Checklist for Artificial Intelligence in Medical Imaging (CLAIM): A Guide for Authors and Reviewers. Radiology: Artificial Intelligence 2020;2:e200029.

8.	Moons KGM, Damen JAA, Kaul T et al. PROBAST+AI: an updated quality, risk of bias, and applicability assessment tool for prediction models using regression or artificial intelligence methods. BMJ 2025;388:e082505.

9.	Elvidge J, Hawksworth C, Avşar TS et al. Consolidated Health Economic Evaluation Reporting Standards for Interventions That Use Artificial Intelligence (CHEERS-AI). Value Health 2024;27:1196-1205.

10.	Norgeot B, Quer G, Beaulieu-Jones BK et al. Minimum information about clinical artificial intelligence modeling: the MI-CLAIM checklist. Nature Medicine 2020;26:1320-1324.

11.	Hernandez-Boussard T, Bozkurt S, Ioannidis JPA, Shah NH. MINIMAR (MINimum Information for Medical AI Reporting): Developing reporting standards for artificial intelligence in health care. Journal of the American Medical Informatics Association 2020;27:2011-2015.

12.	Cacciamani GE, Chu TN, Sanford DI et al. PRISMA AI reporting guidelines for systematic reviews and meta-analyses on AI in healthcare. Nature Medicine 2023;29:14-15.

13.	Lambin P, Leijenaar RTH, Deist TM et al. Radiomics: the bridge between medical imaging and personalized medicine. Nature Reviews Clinical Oncology 2017;14:749-762.

14.	Lekadir K, Frangi AF, Porras AR et al. FUTURE-AI: international consensus guideline for trustworthy and deployable artificial intelligence in healthcare. Bmj 2025;388:e081554.

15.	WHO. Ethics and governance of artificial intelligence for health. Guidance on large multi-modal models. <https://www> who int/publications/i/item/9789240084759/ 2024.

16.	Yeung K. Recommendation of the council on artificial intelligence (OECD). International legal materials 2020;59:27-34.

17.	Ulloa-Cerna AE, Jing L, Pfeifer JM et al. rECHOmmend: An ECG-Based Machine Learning Approach for Identifying Patients at Increased Risk of Undiagnosed Structural Heart Disease Detectable by Echocardiography. Circulation 2022;146:36-47.

18.	Ouyang D, He B, Ghorbani A et al. Video-based AI for beat-to-beat assessment of cardiac function. Nature 2020;580:252-256.

19.	Ahmad T, Lund LH, Rao P et al. Machine Learning Methods Improve Prognostication, Identify Clinically Distinct Phenotypes, and Detect Heterogeneity in Response to Therapy in a Large Cohort of Heart Failure Patients. J Am Heart Assoc 2018;7.

20.	Christensen M, Vukadinovic M, Yuan N, Ouyang D. Vision-language foundation model for echocardiogram interpretation. Nat Med 2024;30:1481-1488.

21.	Vukadinovic M, Tang X, Yuan N et al. EchoPrime: A Multi-Video View-Informed Vision-Language Model for Comprehensive Echocardiography Interpretation. arXiv 2024.

22.	Shankar SV, Dhingra LS, Aminorroaya A et al. Automated Transformation of Unstructured Cardiovascular Diagnostic Reports into Structured Datasets Using Sequentially Deployed Large Language Models. medRxiv 2024.

23.	Tanno R, Barrett DGT, Sellergren A et al. Collaboration between clinicians and vision-language models in radiology report generation. Nat Med 2025;31:599-608.

24.	Blankemeier L, Cohen JP, Kumar A et al. Merlin: A Vision Language Foundation Model for 3D Computed Tomography. Res Sq 2024.

**Supplementary References for Panels 1-9 from Main Manuscript**

1.	Yamashita R, Nishio M, Do RKG, Togashi K. Convolutional neural networks: an overview and application in radiology. Insights Imaging 2018;9:611-629.

2.	Rawshani A, Rawshani A, Smith G et al. Integrating deep learning with ECG, heart rate variability and demographic data for improved detection of atrial fibrillation. Open Heart 2025;12.

3.	Sakli N, Ghabri H, Soufiene BO et al. ResNet-50 for 12-Lead Electrocardiogram Automated Diagnosis. Comput Intell Neurosci 2022;2022:7617551.

4.	Mhamdi L, Dammak O, Cottin F, Dhaou IB. Artificial Intelligence for Cardiac Diseases Diagnosis and Prediction Using ECG Images on Embedded Systems. Biomedicines 2022;10.

5.	Ferreira DL, Lau C, Salaymang Z, Arnaout R. Self-supervised learning for label-free segmentation in cardiac ultrasound. Nat Commun 2025;16:4070.

6.	Liang B, Peng F, Luo D et al. Automatic segmentation of 15 critical anatomical labels and measurements of cardiac axis and cardiothoracic ratio in fetal four chambers using nnU-NetV2. BMC Med Inform Decis Mak 2024;24:128.

7.	Hinck D, Segeroth M, Miazza J et al. Automatic Segmentation of Cardiovascular Structures on Chest CT Data Sets: An Update of the TotalSegmentator. Eur J Radiol 2025;185:112006.

8.	Schilling M, Unterberg-Buchwald C, Lotz J, Uecker M. Assessment of deep learning segmentation for real-time free-breathing cardiac magnetic resonance imaging at rest and under exercise stress. Sci Rep 2024;14:3754.

9.	Ansari Y, Mourad O, Qaraqe K, Serpedin E. Deep learning for ECG Arrhythmia detection and classification: an overview of progress for period 2017-2023. Front Physiol 2023;14:1246746.

10.	Skandarani Y, Lalande A, Afilalo J, Jodoin PM. Generative Adversarial Networks in Cardiology. Can J Cardiol 2022;38:196-203.

11.	Yoon S, Nakamori S, Amyar A et al. Accelerated Cardiac MRI Cine with Use of Resolution Enhancement Generative Adversarial Inline Neural Network. Radiology 2023;307:e222878.

12.	Hampe N, van Velzen SGM, Wolterink JM et al. Graph neural networks for automatic extraction and labeling of the coronary artery tree in CT angiography. J Med Imaging (Bellingham) 2024;11:034001.

13.	Xu H, Wu Y. G2ViT: Graph Neural Network-Guided Vision Transformer Enhanced Network for retinal vessel and coronary angiograph segmentation. Neural Netw 2024;176:106356.

14.	Lomoio U, Vizza P, Giancotti R et al. A convolutional autoencoder framework for ECG signal analysis. Heliyon 2025;11:e41517.

15.	Staffini A, Svensson T, Chung UI, Svensson AK. A Disentangled VAE-BiLSTM Model for Heart Rate Anomaly Detection. Bioengineering (Basel) 2023;10.

16.	Al-Hammuri K, Gebali F, Kanan A, Chelvan IT. Vision transformer architecture and applications in digital health: a tutorial and survey. Vis Comput Ind Biomed Art 2023;6:14.

17.	Vaid A, Jiang J, Sawant A et al. A foundational vision transformer improves diagnostic performance for electrocardiograms. NPJ Digit Med 2023;6:108.

18.	Christensen M, Vukadinovic M, Yuan N, Ouyang D. Vision-language foundation model for echocardiogram interpretation. Nat Med 2024;30:1481-1488.

19.	Vukadinovic M, Tang X, Yuan N et al. EchoPrime: A Multi-Video View-Informed Vision-Language Model for Comprehensive Echocardiography Interpretation. arXiv 2024.

20.	Arnaout R. Adapting vision-language AI models to cardiology tasks. Nat Med 2024;30:1245-1246.

21.	Shankar SV, Dhingra LS, Aminorroaya A et al. Automated Transformation of Unstructured Cardiovascular Diagnostic Reports into Structured Datasets Using Sequentially Deployed Large Language Models. medRxiv 2024.

22.	Tanno R, Barrett DGT, Sellergren A et al. Collaboration between clinicians and vision-language models in radiology report generation. Nat Med 2025;31:599-608.

23.	Blankemeier L, Cohen JP, Kumar A et al. Merlin: A Vision Language Foundation Model for 3D Computed Tomography. Res Sq 2024.

24.	Lu MY, Chen B, Williamson DFK et al. A multimodal generative AI copilot for human pathology. Nature 2024;634:466-473.

25.	Moore A, Bell M. XGBoost, A Novel Explainable AI Technique, in the Prediction of Myocardial Infarction: A UK Biobank Cohort Study. Clin Med Insights Cardiol 2022;16:11795468221133611.

26.	Arik SO, Pfister T. TabNet: Attentive Interpretable Tabular Learning. arXiv 2019.

27.	Nguyen HV, Byeon H. Prediction of Out-of-Hospital Cardiac Arrest Survival Outcomes Using a Hybrid Agnostic Explanation TabNet Model. Mathematics 2023;11.

28.	Behnam V, Chern TL, Wiedenhaupt J, Shimbo D, Schwartz JE, Sia SK. Assessment of cardiovascular risk from ambulatory blood pressure measurements enabled by deep learning. npj Biosensing 2025;2.

29.	Titar RR, Ramanathan M. Variational autoencoders for generative modeling of drug dosing determinants in renal, hepatic, metabolic, and cardiac disease states. Clin Transl Sci 2024;17:e13872.

30.	Alnasser TN, Abdulaal L, Maiter A et al. Advancements in cardiac structures segmentation: a comprehensive systematic review of deep learning in CT imaging. Front Cardiovasc Med 2024;11:1323461.

31.	Bernard O, Lalande A, Zotti C et al. Deep Learning Techniques for Automatic MRI Cardiac Multi-Structures Segmentation and Diagnosis: Is the Problem Solved? IEEE Trans Med Imaging 2018;37:2514-2525.

32.	Leclerc S, Smistad E, Pedrosa J et al. Deep Learning for Segmentation Using an Open Large-Scale Dataset in 2D Echocardiography. IEEE Trans Med Imaging 2019;38:2198-2210.

33.	Zhou H, Hu B, Yi N, Li Q, Ergu D, Liu F. Balancing High-performance and Lightweight: HL-UNet for 3D Cardiac Medical Image Segmentation. Acad Radiol 2024;31:4340-4351.

34.	Cui H, Yuwen C, Jiang L, Xia Y, Zhang Y. Multiscale attention guided U-Net architecture for cardiac segmentation in short-axis MRI images. Comput Methods Programs Biomed 2021;206:106142.

35.	Islam MR, Qaraqe M, Serpedin E. CoST-UNet: Convolution and swin transformer based deep learning architecture for cardiac segmentation. Biomedical Signal Processing and Control 2024;96.

36.	Vafaeezadeh M, Behnam H, Gifani P. Ultrasound Image Analysis with Vision Transformers-Review. Diagnostics (Basel) 2024;14.

37.	Fakhfakh M, Sarry L, Clarysse P. HALSR-Net: Improving CNN Segmentation of Cardiac Left Ventricle MRI with Hybrid Attention and Latent Space Reconstruction. Comput Med Imaging Graph 2025;123:102546.

38.	Suha KT, Lubenow H, Soria-Zurita S, Haw M, Vettukattil J, Jiang J. The Artificial Intelligence-Enhanced Echocardiographic Detection of Congenital Heart Defects in the Fetus: A Mini-Review. Medicina (Kaunas) 2025;61.

39.	Zhao M, Wei Y, Wong KKL. A Generative Adversarial Network technique for high-quality super-resolution reconstruction of cardiac magnetic resonance images. Magn Reson Imaging 2022;85:153-160.

40.	Streiffer CD, Levin MG, Witschey WR, Anyanwu EC. Denoising diffusion model for increased performance of detecting structural heart disease. medRxiv 2024.

41.	Nazi ZA, Peng W. Large Language Models in Healthcare and Medical Domain: A Review. Informatics 2024;11:57.

42.	Neveditsin N, Lingras P, Mago V. Clinical insights: A comprehensive review of language models in medicine. PLOS Digit Health 2025;4:e0000800.

43.	Chao CJ, Banerjee I, Arsanjani R et al. Evaluating large language models in echocardiography reporting: opportunities and challenges. Eur Heart J Digit Health 2025;6:326-339.

44.	Subramaniam S, Rizvi S, Ramesh R et al. Ontology-guided machine learning outperforms zero-shot foundation models for cardiac ultrasound text reports. Sci Rep 2025;15:5456.

45.	Zhou Y, Ong H, Kennedy P et al. Evaluating GPT-V4 (GPT-4 with Vision) on Detection of Radiologic Findings on Chest Radiographs. Radiology 2024;311:e233270.

46.	Bannur S, Bouzid K, Castro DC et al. MAIRA-2: Grounded Radiology Report Generation. arXiv 2024.

47.	Hessel J, Holtzman A, Forbes M, Bras RL, Choi Y. CLIPScore: A Reference-free Evaluation Metric for Image Captioning. arXiv 2022.

48.	Jin R, Hou T, Jin T, Yuan J, Du C. A method for image–text matching based on semantic filtering and adaptive adjustment. EURASIP Journal on Image and Video Processing 2024.

49.	Priya S, Dhruba DD, Sorensen E et al. ComBat Harmonization of Myocardial Radiomic Features Sensitive to Cardiac MRI Acquisition Parameters. Radiol Cardiothorac Imaging 2023;5:e220312.

50.	Fathi MF, Perez-Raya I, Baghaie A et al. Super-resolution and denoising of 4D-Flow MRI using physics-Informed deep neural nets. Comput Methods Programs Biomed 2020;197:105729.

51.	Simon BD, Ozyoruk KB, Gelikman DG, Harmon SA, Turkbey B. The future of multimodal artificial intelligence models for integrating imaging and clinical metadata: a narrative review. Diagn Interv Radiol 2024.

52.	Chawla NV, Bowyer KW, Hall LO, Kegelmeyer WP. SMOTE: Synthetic Minority Over-sampling Technique. Journal of Artificial Intelligence Research 2002;16.

53.	Yi X, Walia E, Babyn P. Generative adversarial network in medical imaging: A review. Med Image Anal 2019;58:101552.

54.	Kazerouni A, Aghdam EK, Heidari M et al. Diffusion models in medical imaging: A comprehensive survey. Med Image Anal 2023;88:102846.

55.	Thirunavukarasu AJ, Ting DSJ, Elangovan K, Gutierrez L, Tan TF, Ting DSW. Large language models in medicine. Nat Med 2023;29:1930-1940.

56.	Singhal K, Azizi S, Tu T et al. Large language models encode clinical knowledge. Nature 2023;620:172-180.

57.	Comeau DS, Bitterman DS, Celi LA. Preventing unrestricted and unmonitored AI experimentation in healthcare through transparency and accountability. NPJ Digit Med 2025;8:42.

58.	Vaswani A, Shazeer N, Parmar N et al. Attention Is All You Need. Proceedings of the 31st International Conference on Neural Information Processing Systems (NIPS), 2017, 2017.

59.	Seoni S, Jahmunah V, Salvi M, Barua PD, Molinari F, Acharya UR. Application of uncertainty quantification to artificial intelligence in healthcare: A review of last decade (2013-2023). Comput Biol Med 2023;165:107441.

60.	Gawlikowski J, Tassi CRN, Ali M et al. A survey of uncertainty in deep neural networks. Artificial Intelligence Review 2023;56:1513–1589.

61.	Hüllermeier E, Waegeman W. Aleatoric and epistemic uncertainty in machine learning: an introduction to concepts and methods. Machine Learning 2021;110:457–506.

62.	Hanneman K, Picano E, Campbell-Washburn AE et al. Society for Cardiovascular Magnetic Resonance recommendations toward environmentally sustainable cardiovascular magnetic resonance. J Cardiovasc Magn Reson 2025:101840.
